# Characterization of the Ubiquitin-Conjugating Enzyme Gene Family in Rice and Evaluation of Expression Profiles under Abiotic Stresses and Hormone Treatments

**DOI:** 10.1371/journal.pone.0122621

**Published:** 2015-04-22

**Authors:** Zhiguo E, Yuping Zhang, Tingting Li, Lei Wang, Heming Zhao

**Affiliations:** 1 China National Rice Research Institute, 359 Tiyuchang Road, Hangzhou, 310006, China; 2 Center for Genomics and Biotechnology, Fujian Agriculture and Forestry University, Fuzhou, 350002, Fujian Province, China; University of Delhi South Campus, INDIA

## Abstract

Ubiquitin-conjugating enzyme E2s (UBCs), which catalyze the transfer of ubiquitin to substrate or E3 ligases, are key enzymes in ubiquitination modifications of target proteins. However, little is known about the knowledge of UBC gene family in rice. In this study, a total of 39 UBC encoding genes, which all contained an UBC domain with a cysteine active site, were identified in the rice genome. These were classified into fifteen distinct subfamilies based upon their sequence similarity and phylogenetic relationships. A subset of 19 *OsUBC* genes exhibited chromosomal duplication; 4 and 15 *OsUBC* genes were tandemly and segmentally duplicated, respectively. Comprehensive analyses were performed to investigate the expression profiles of *OsUBC* genes in various stages of vegetative and reproductive development using data from EST, Microarrays, MPSS, and real-time PCR. Many *OsUBC* genes exhibited abundant and tissue-specific expression patterns. Moreover, 14 *OsUBCs* were found to be differentially expressed under treatments with drought, or salt stresses. The expression analysis after treatments with IAA, 6-BA, GA and ABA indicated that almost all *OsUBC* genes were responsive to at least two of the four hormones. Several genes were significantly down-regulated under all of the hormone treatments, and most of the genes reduced by 6-BA were also reduced by GA. This study will facilitate further studies of the *OsUBC* gene family and provide useful clues for functional validation of *OsUBCs* in rice.

## Introduction

Ubiquitination is the crucial regulatory step for the selective protein degradation mechanism in the ubiquitin-26S proteasome pathway in plants [1–3]. Ubiquitination is known to regulate important functions in a wide variety of plant growth and developmental processes including photomorphogenesis, vascular differentiation, flower development, both phytohormone and light signaling, as well as biotic and abiotic stress responses [4–7]. The key enzymes involved in ubiquitination canonically include an E1 (ubiquitin-activating enzyme, UBA), an E2 (ubiquitin-conjugating enzyme, UBC), and an E3 (ubiquitin-ligating enzyme). E2 UBC enzymes catalyze the transfer of the activated ubiquitin to the substrates or the E3 enzymes [8], mediate the formation of polyubiquitin chains on target proteins [9], and together with E3 determine substrate specificity in the ubiquitination system [10].

UBCs exist very widely in eukaryotic cells, and have a conserved ~150-residue so-called “UBC domain” that harbors the active site cysteine residue required for enzyme-ubiquitin thioester formation [11–12]. On the N-terminal side of the active cysteine residue, there are several highly conserved motifs, including ‘HPN’ and ‘PxxPP’ [12]. Based on the conserved UBC domain, E2 isoforms (UBCs) have been identified in the genomes of eukaryotes, for example, there are 13 members that have been identified in *Saccharomyces cerevisiae* [12], 37 in *Arabidopsis thaliana* [13], 20 in *C*. *elegans* [14], and 37 in human [15]. Recently, 48 E2 UBCs were identified in rice genome and grouped into 15 different groups [10].

To date, our knowledge about the role of E2s (UBCs) in plants is still relatively limited, especially as compared to that of E3s. Some E2 enzymes possess additional N- and/or C- terminal extensions that are involved in substrate specificity and intracellular localization of the enzyme [16]. Among the 37 E2s in Arabidopsis, 17 proteins had been detected to possess E2 catalytic activity *in vitro*; examples of these included AtUBC1, AtUBC2, AtUBC13, and AtUBC32 [13, 17]. Further functional analyses revealed that AtUBC1 and AtUBC2 were involved in leaf development, activation of floral repressor genes, and UV stress tolerance responses. The loss-of-function double mutant *atubc1-1atubc2-1* showed a dramatically reduced number of rosette leaves and an early-flowering phenotype [18]. AtUBC24 regulated inorganic phosphate uptake, allocation and remobilization [19–20], and was a known target gene of miR399s [21]. AtUBC32 was known to play a role in brassinosteroid (BR)-mediated growth promotion and salt stress responses in Arabidopsis [22]. In the wild rice (*Oryza grandiglumis*) genome, the *OgUBC1* gene encoded an ubiquitin-conjugating enzyme that demonstrated typical E2 activity *in vitro*, and was required for cellular responses to biotic and abiotic stresses in plants [23]. CsUBC13 from cucumber (*Cucumis sativus*) was known to mediate the formation of Lys63-linked ubiquitin chains, and played critical roles in epidermal cell differentiation and in Fe deficiency induced developmental responses [24]. In rice, OsUBC5a and OsUBC5b were known to function as E2s and catalyzed the EL5-mediated ubiquitination of target proteins [25]. *Arabidopsis* plants overexpressing *VrUBC1* mung bean (*Vigna radiata*) displayed highly sensitive responses to ABA and osmotic stress during germination, enhanced ABA- or salt-induced stomatal closing, and increased drought stress tolerance [26].

Although the roles of several plant UBCs have been characterized, our understanding of the biological function of the majority of these enzymes is still very limited; this is especially true for the UBCs of food crops such as rice (*Oryza sativa* L.). Rice is the main staple food for a large segment of the world’s population and has become the primary model for cereal species in plant science research. Though the total number of OsUBCs in rice had been characterized and already appropriately named [10], little was known about their expression patterns and response to hormones or abiotic stresses. As such, there is an urgent need for a thorough expression analysis of the *OsUBCs* family. This study provides a global overview of the OsUBCs that harbors the active site cysteine residue in rice, and it contains the gene structures, chromosome locations, phylogeny, and the *OsUBC* mRNAs expression profiling resulting from various organs/tissues of rice and from rice plants treated with abiotic stresses and several plant hormones. These results provide a foundation and will greatly aid in the functional validation of the *UBC* genes in rice and will broaden our understanding of the roles of UBCs in various aspects of vegetative and reproductive development in rice and other crops.

## Materials and Methods

### Plant materials and treatments with abiotic stresses or hormones

In order to evaluate the expression profiles of *OsUBC* genes in various organs at different developmental stages, the rice seedlings of the cultivar *Nipponbare* were grown in the field during the normal growing season at 30–34: 22–26°C (day: night) and 80–95% humidity with a photoperiod of 14 h. The five materials tested in the expression analysis were: (1) 30-day-old roots (R); (2) 60-day-old leaves (L); (3) 90-day-old stems (St); (4) 15–20cm panicles (P); and (5) seeds at the 10 DAP (day after pollination) stage (Sd). For abiotic stress treatments, as described in detail previously [27–28], rice seeds were immersed in water at 37°C for 30 h, and then were sown on a plastic net that was floating on a nutrient solution in a growth chamber at 28°C [light:dark = 14h:10h]. Then, 7-day-old seedlings were carefully transferred onto paper at 28°C as drought stress, placed in 400 mM NaCl solution at 28°C as salt stress, and kept in deionized water for 3h at 4°C as cold stress. For hormone treatments, 7-day-old seedlings were transferred into containers and treated with 10 μM IAA, 10 μM 6-BA, 10 μM GA, or 25 μM ABA and placed in a 28°C illumination incubator. Parallel control samples were prepared by keeping the seedlings in deionized water with the same concentration of ethanol or NaOH used in the treatments. At 3, 6, 12, 24h after these treatments, seedlings were harvested. IAA, GA, and ABA were dissolved in 200 μL ethanol, 6-BA was dissolved in 100 μL NaOH (1mol/L), then they were diluted with 500 mL deionized water for treatments. All materials harvested were immediately frozen in liquid nitrogen and stored at -80°C prior to RNA extraction.

### Database screening and identification of *OsUBCs*

To mine all of the putative UBC members in the rice genome, three approaches were employed, as described in detail previously [28]. Firstly, the key word "ubquitin-conjugating enzyme" was used as a query to search in the Rice Genome Express Database (http://signal.salk.edu/cgi-bin/RiceGE). Secondly, UBC domain (PF00179) searching was performed using the MSU-RGAP website (http://rice.plantbiology.msu.edu/domain_searchs.html). Thirdly, protein sequences of putative OsUBCs were downloaded from Interpro searches (http://www.ebi.ac.uk/interpro/ISearch?Query=PF00179); The resulting protein sequences were then used as queries to perform database searches against both MSU-RGAP (http://rice.plantbiology.msu.edu/) and NCBI (http://www.ncbi.nlm.nih.gov/). After removing the redundant sequences, the remaining protein sequences which contained the characteristic active cysteine residue were defined as putative ubquitin-conjugating enzymes. The full-length cDNA accessions, coding sequence lengths, the gene structure for each gene, and the characteristics of proteins were obtained from KOME (http://cdna01.dna.affrc.go.jp/cDNA/) and MSU-RGAP (http://rice.plantbiology.msu.edu/). The Gene structures of the *OsUBCs* were analyzed using the GSDS (Gene Structure Display Server) website (http://gsds.cbi.pku.edu.cn/).

### Chromosomal localization and gene duplication

The physical positions of *OsUBCs* were used to map these genes onto the corresponding rice chromosomes. The distribution of *OsUBC* genes on chromosomes was drawn by using MapChart software [29]. *OsUBC* genes on duplicated chromosomal segments were explored by searching the segmental genome duplication of rice with the RGAP segmental duplication database (http://rice.plantbiology.msu.edu/segmental_dup/500kb/segdup_500kb.shtml). Genes distributed and separated by five or fewer genes were considered to be tandem duplicates.

### Phylogenetic analysis and sequence alignment

Multiple sequence alignment was performed using ClustalX version 1.83. An unrooted phylogenetic tree based on the full-length protein sequences of the OsUBCs was constructed using the neighbor-joining method and Mrbayes program [27]. MEGA software version 4 [30] was used to display the phylogenetic tree. Using the same method, a combined tree with both OsUBCs and AtUBCs was generated. The sequence conservation of the UBC domains in OsUBCs was analyzed using DNAMAN software [28]. The molecular modelling of OsUBC proteins were conducted by using SWISS-MODEL (http://www.swissmodel.expasy.org/), and the 3D structures of the typical members were displayed with PyMOL software [31–33].

### Digital expression analysis and qRT-PCR of *OsUBCs*

Expression analysis of *OsUBC* genes using EST, microarray, and MPSS expression data was performed as described by Ma et al (2011). Additionally, the absolute values of *AtUBCs* comparable to those used with rice were also downloaded from TAIL website (http://www.arabidopsis.org/) (S4 and S6 Tables). To make the absolute signal values suitable for cluster display, these absolute signal values were divided by the average of all of the absolute values. Cluster and Treeview software [27] were used to generate hierarchical cluster display using the logarithmic values of the ratios from the previous step. In the comparative expression analysis of *OsUBCs* and *AtUBCs*, the genes that were up- or down-regulated by at least two-fold were considered to be differentially expressed.

As described in detail previously [28], the total RNA extraction from various rice tissues, cDNA synthesis and quantitative RT-PCR were performed to analyze *OsUBC* genes expression. Rice ubiquitin 5 (*UBQ5*, LOC_Os03g13170) was used as an internal control gene [34]. The gene-specific primers of *OsUBCs* were listed in S5 Table. The relative expression levels were evaluated using the comparative *C*_*T*_ method. The following equation was used to calculate the genes expression in different samples: Fold change = 2^-ΔΔ*C*^ *T* = [(*C*_*T*_ gene of interest—*C*_*T*_ internal control) sample A—(*C*_*T*_ gene of interest—*C*_*T*_ internal control) sample B] [35].

## Results

### Identification of *UBC* genes in rice genome

We used multiple bioinformatics resources in our efforts to comprehensively explore the entire *UBC* gene family in rice. 49 protein sequences were obtained through UBC domain searching (the ubiquitin-conjugating_E2 family, PF00179) with the MSU-RGAP (MSU-Rice Genome Annotation Project) database (http://rice.plantbiology.msu.edu/analyses_search_domain.shtml). 90 protein sequences of OsUBCs from *japonica* were deposited in InterPro of the European Bioinformatics Institute database (http://www.ebi.ac.uk/interpro/). In the Rice Genome Express Database (http://signal.salk.edu/cgi-bin/RiceGE), using a keyword search for "ubiquitin-conjugating enzyme", 53 genes were identified. The sequences obtained with these analyses/searches were used as queries in BLAST searches against the rice genome entries in both the MSU-RGAP and the NCBI databases. Following removal of the sequence redundancies and eliminating alternate splice variants of the same gene, we initially identified 50 putative *UBC* genes in rice. Eleven candidates were excluded from further analysis because they contained incomplete UBC domains that lacked the cysteine active site. A total of 39 *UBCs* were thus identified in rice. The detailed information about each gene locus, FL-cDNA, ORF length for each *OsUBC*, and characteristics of corresponding proteins are detailed in Table 1.

**Table 1:** The general information and sequence characterization of 39 *OsUBC* genes.

Gene^a^	Accession Number		ORF^d^	Protein^e^			Sub^f^	Expression^g^
	RGAP Locus^b^	KOME^c^	(bp)	Size(aa)	MW(D)	pI		
*OsUBC1*	LOC_Os10g39120	AK121221	480	160	18045.6	8.79	I	A BC D
*OsUBC2*	LOC_Os03g03130	AK062161	480	160	18052.6	8.47	I	A B C D
*OsUBC3*	LOC_Os04g49130	AK121581	480	160	18085.6	8.79	I	A B C D
*OsUBC4*	LOC_Os10g11260	AK064839	549	183	20538.6	8.77	II	A B C D
*OsUBC5*	LOC_Os08g28680	AK070541	549	183	20695.7	8.46	II	A B C D
*OsUBC6*	LOC_Os09g15320	AK073821	549	183	20772.9	8.79	II	A B C D
*OsUBC7*	LOC_Os07g07240	AK070524	456	152	17311.5	5.75	III	A B C D
*OsUBC8*	LOC_Os05g08960	AK112003	456	152	17305.6	5.20	III	A B C D
*OsUBC9*	LOC_Os03g57790	AK067703	456	152	17309.6	5.75	III	A B C D
*OsUBC10*	LOC_Os10g31000	AK066993	549	183	20798.4	4.01	IV	A B C D
*OsUBC11*	LOC_Os01g62244	AK112030	507	169	19013.6	5.24	V	A B C D
*OsUBC12*	LOC_Os05g38550	AK111857	630	210	23433.7	5.73	V	A B C D
*OsUBC13*	LOC_Os02g02830	AK120237	444	148	16600.2	7.96	VI	A B C D
*OsUBC14*	LOC_Os01g46926	AK063826	441	147	16573.1	8.08	VI	A B D
*OsUBC15*	LOC_Os02g16040	AK121248	444	148	6446.0	8.07	VI	A B C D
*OsUBC16*	LOC_Os04g57220	AK119682	444	148	16507.0	8.07	VI	A B C D
*OsUBC17*	LOC_Os06g30970	AK066232	444	148	16391.9	8.07	VI	A B C D
*OsUBC18*	LOC_Os09g12230	AK065902	444	148	16603.0	7.57	VI	A B C D
*OsUBC22*	LOC_Os01g60410	AK058360	444	148	16517.1	8.07	VI	A B C D
*OsUBC23*	LOC_Os01g60360	NA	444	148	16517.1	8.07	VI	D
*OsUBC25*	LOC_Os03g47770	AK103809	483	161	18297.8	8.33	VII	A B C D
*OsUBC26*	LOC_Os12g44000	AK071648	483	161	18346.8	8.32	VII	A B C D
*OsUBC27*	LOC_Os01g16650	AK105320	573	191	20199.6	5.68	VIII	A B C D
*OsUBC32*	LOC_Os02g42314	AK059694	489	163	18296.2	6.34	IX	A B D
*OsUBC33*	LOC_Os06g45000	AK058614	756	252	27433.4	9.74	X	A B C D
*OsUBC34*	LOC_Os01g03520	AK103631	3189	1063	117135	4.26	XI	A B C D
*OsUBC35*	LOC_Os05g48390	NA	2628	876	96800.5	4.43	XI	A B C D
*OsUBC36*	LOC_Os05g06120	NA	3135	1045	109484	5.39	XI	C
*OsUBC37*	LOC_Os01g13280	AK100714	1122	374	41918.5	9.07	XI	A B C D
*OsUBC39*	LOC_Os01g48580	NA	1479	493	55119.4	6.50	XI	C
*OsUBC40*	LOC_Os09g12310	AK107258	1119	373	41928.8	9.14	XI	A B C D
*OsUBC41*	LOC_Os05g48380	AK121717	1527	509	56867.9	4.90	XI	A B C D
*OsUBC42*	LOC_Os01g13170	AK067926	1455	485	53830.3	4.55	XI	A B C D
*OsUBC43*	LOC_Os05g14300	AK069430	1401	467	51947.4	4.53	XI	A B C D
*OsUBC44*	LOC_Os01g70140	AK073357	585	195	21323.2	4.71	XII	A B C D
*OsUBC45*	LOC_Os03g19500	AK100355	939	313	34711.4	6.81	XIV	A B C D
*OsUBC46*	LOC_Os06g09330	AK065085	720	240	26989.3	8.91	XIV	A B C D
*OsUBC47*	LOC_Os01g48280	AK122067	459	153	17159.8	7.51	XV	A B C D
*OsUBC48*	LOC_Os01g42040	AK063933	1668	556	60665.4	9.58	XVI	A B C

^*a*^ Systematic designation given to rice *UBCs* in this study.

^*b*^ Locus identity number of *OsUBCs* assigned by RGAP.

^*c*^ Full-length cDNA accession number of *OsUBCs* obtained from KOME.

^*d*^ Length of the open reading frame for *OsUBCs*.

^*e*^ Protein characterization of OsUBCs obtained from RGAP.

^*f*^ Subfamily of OsUBCs by Phylogenetic relationship.

^*g*^ Evidence for gene expression from (A) full-length cDNA, (B) ESTs, (C) microarray data, (D) massively parallel signature sequencing (MPSS).

ORF, open reading frame; bp, base pair; aa, amino acids; MW, molecular weight; pI, isoelectric point; Sub, subfamily; NA, not available.

By using GSDS website (Gene Structure Display Server) (http://gsds.cbi.pku.edu.cn/), the comparison of the full-length cDNA sequences and the corresponding genomic DNA sequences was performed to determine the numbers and positions of exons and introns of each *OsUBC* gene. Only one gene (*OsUBC27*) lacked introns; the number of introns in the coding sequences of the other 38 genes ranged from one to eight (S1 Fig). Three *OsUBC* genes (*OsUBC23/36/*39) had no untranslated regions. Within a given subfamily, most members tended to share similar intron/exon structure and gene length. For example, the three members (*OsUBC1/2/*3) of subfamily I each contain four introns and five exons, and are all nearly 3500 bp in length (S1 Fig).

### Chromosomal localization and gene duplication

**Fig 1:**
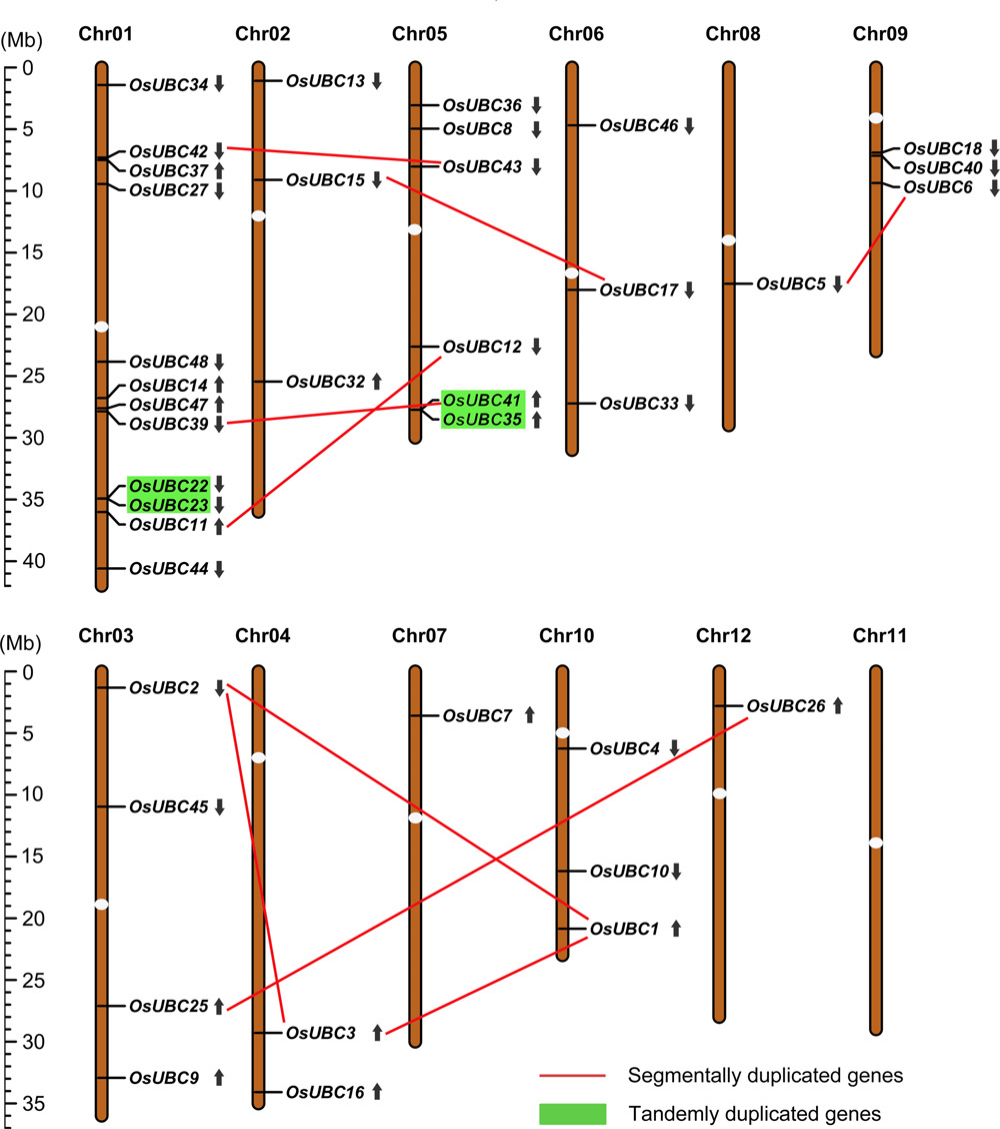
Chromosomal localization and gene duplication events of *OsUBC* genes. Respective chromosome numbers are indicated at the top of each bar. The scale on the left is in megabases (Mb). The white ellipses on the chromosomes (vertical bars) indicate the position of centromeres. The chromosome order is arranged to bring duplicated regions in the vicinity. The segmental duplication genes are connected by straight line in color. The tandemly duplicated genes are shaded with rectangles in light green. The arrows next to gene names show the direction of transcription.

Based on the RGAP loci coordinates (http://rice.plant biology.msu.edu/cgi-bin/gbrowse/rice/#search), the exact locations and orientation of the *OsUBC* genes on the rice chromosomes were determined. The 39 *OsUBC* genes were distributed across 11 rice chromosomes, and there was no substantial clustering of *OsUBC* genes in any particular rice chromosome. The densities of *OsUBC* genes were relatively higher on specific chromosomes such as chromosome 1 and 5, particularly the long arm of chromosome 1. In contrast, several large chromosomal regions are devoid of *OsUBC* genes, such as in the short arm of chromosomes 4, 8, 9, and 10, and in the long arm of chromosomes 7 and 12. Rice chromosome 1 has twelve UBC genes, chromosome 5 has six, and chromosome 3 has four. Chromosomes 2, 6, 9, and 10 each have three UBC genes, chromosome 4 has two and chromosomes 7, 8, and 12 each only contain one UBC gene (Fig 1).

To explore how duplication events during evolution have impacted the expansion of the *OsUBC* family, we investigated segmental and tandem duplications. Among the 39 *OsUBC* genes, 48.72% (19 of 39) were involved in duplication events, including 15 genes with segmental duplication and 4 genes with tandem duplication. The 15 *OsUBC* genes could be assigned to the duplicated segmental regions of rice chromosomes mapped by the MSU-RGAP segmental duplication database when the maximal length between collinear gene pairs was 500 kb. According to the criterion of separation by less than 5 intervening genes and ≥ 50% homology at protein level, a total of 4 genes were found to be tandemly duplicated; these fell into two groups. The tandemly duplicated genes were on chromosomes 1 and 5 (Fig 1). Interestingly, three genes (*OsUBC35/39/41*) were expanded through both segmental and tandem duplications. Moreover, most of the duplicated genes exhibited high sequence similarity in UBC domains. These results showed that the gene duplication events contributed to the expansion of the *OsUBC* family in rice.

### Phylogenetic analysis and multiple sequence alignment

**Fig 2:**
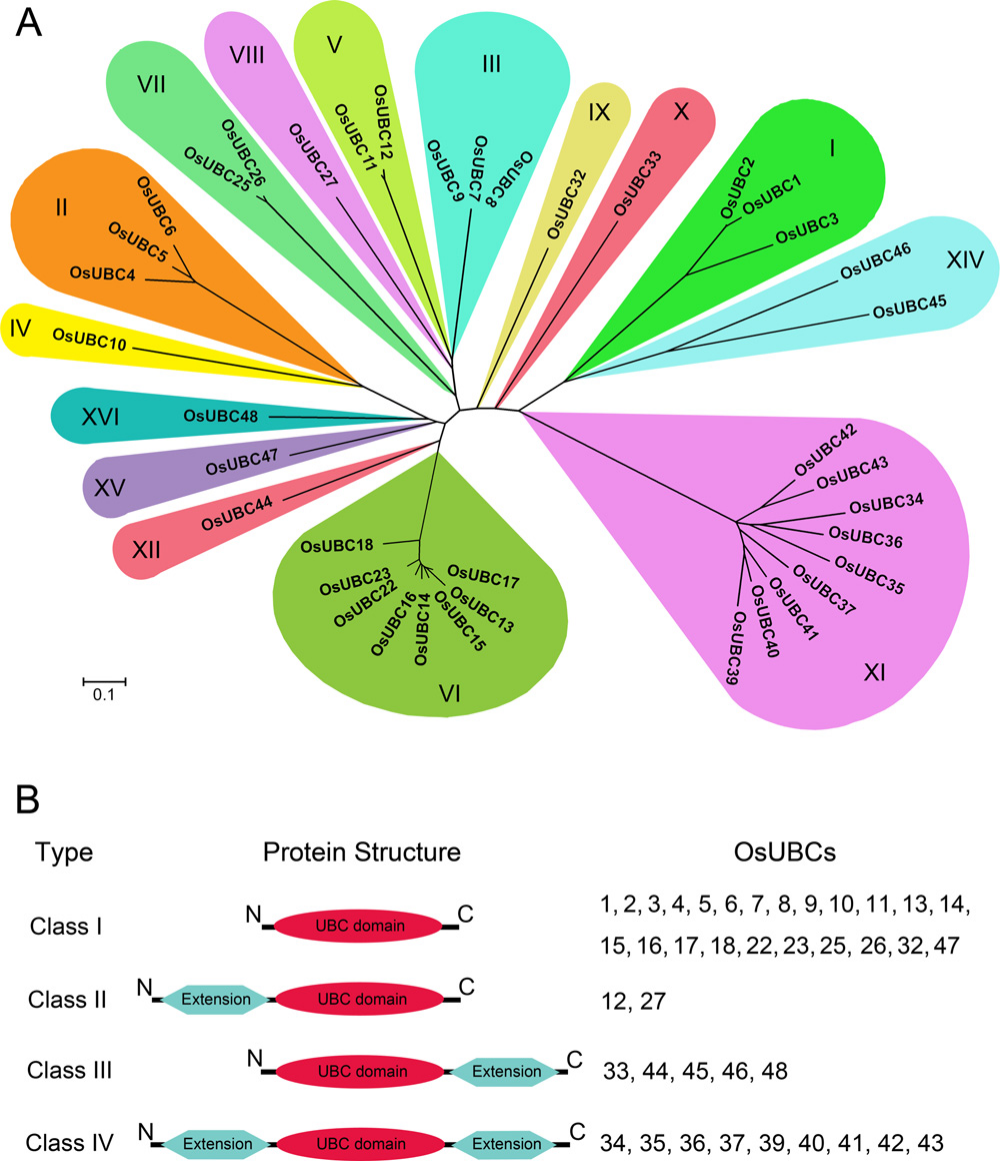
Phylogenetic relationship and protein types of OsUBCs. Phylogenetic relationship of OsUBCs. Scale bar represents 0.1 amino acid substitution per site. The proteins on the tree can be divided into fifteen distinct subfamilies. The branches of different subfamilies are marked by different colors. (B) Protein types of OsUBCs. The numbers mean proteins encoded by corresponding *OsUBCs*.

To examine in detail the phylogenetic relationship and functional divergence among the 39 OsUBCs in rice, a phylogenetic tree was constructed from alignments of the full-length sequences of the UBC proteins using both neighbour-joining methods and Bayesian methods in the MrBayes program. The phylogenetic trees resulting from both of these analyses were very similar (Fig 2A). The 39 OsUBCs were classified into 15 distinct subfamilies; the subfamilies were named according to their identity to *Arabidopsis* UBC proteins and included subfamilies I–XII and subfamilies XIV–XVI. The rice homolog of the group XIII Arabidopsis UBC31 E2 was not present. The proteins in subfamilies I and II of *Arabidopsis* lack the active-site cysteine residue and belong to ubiquitin-conjugating E2 enzyme variant UEV [16]; the UEV proteins in rice were excluded from this study. Among the 15 OsUBC subfamilies, subfamily XI with nine OsUBCs was the largest. There were eight OsUBCs in subfamily VI, as is the case in *Arabidopsis*. Subfamilies IV, VIII, IX, X, XII, XV, and XVI each contained only a single member (Fig 2A).

In order to identify orthologous genes between rice and *Arabidopsis*, a combined phylogenetic tree with OsUBCs and AtUBCs was generated (S2 Fig). In this analysis, similar subfamilies were formed as compared with the OsUBC tree. Except for the subfamily XIII, each clade of a distinct subfamily consisted of UBCs from both rice and *Arabidopsis*, and the total number of *OsUBCs* and *AtUBC*s in most *UBC* subfamily was also similar. The UBC orthologs between rice and *Arabidopsis* in subfamily III, VIII, IX, X, XII, and XVI showed very high sequence identities. These results indicated that the formation of UBC family in rice and *Arabidopsis* occurred before the split of monocots and eudicots.

Based on the presence of the N- and/or C- terminal extensions that are typically responsible for the functional differences between E2s, the UBCs are divided into four types [13, 36–37]. Class I UBCs consist of only the catalytic domain. Classes II UBCs have N-terminal extensions, class III UBCs have C-terminal extensions. Class IV UBCs have the both N- and C- extensions [14]. Our analysis showed that there were twenty-three Class I OsUBC proteins, two Class II OsUBCs, five Class III OsUBCs, and nine Class IV OsUBCs (Fig 2B). 3D structure of the typical member of each class was displayed in the S3 Fig. These proteins in class I, only containing UBC domain, was ranging from 147–183aa, class II from 191–210aa, class III from 195–556aa, and class IV from 373–1063aa (Table 1).

**Fig 3:**
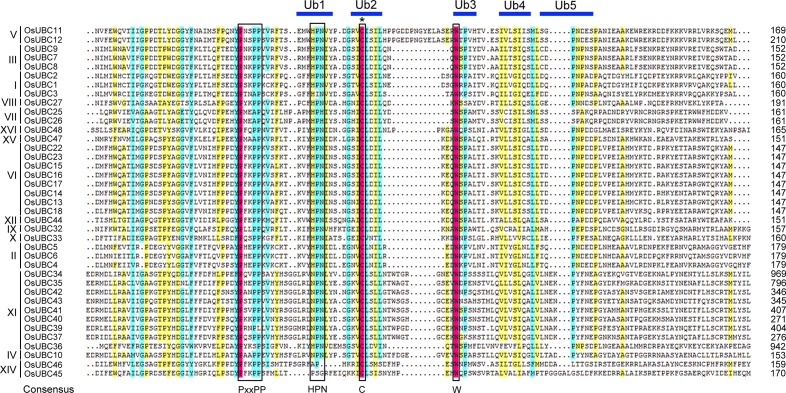
Multiple sequence alignment of UBC domain in OsUBCs. Identical, conservative and block of similar amino acid residues are shaded in red, light blue and yellow, respectively. The asterisk symbol above the alignment indicates the conserved active-site cysteine of UBCs. The conserved motifs are bordered (black rectangles), including PxxPP motif and HPN tripeptide motif. Ubiquitin thioester intermediate interaction residues are marked by blue lines and shown as Ub1-5.

To clearly understand the sequence characteristics of OsUBCs, we conducted a multiple sequence alignment using the deduced amino acid sequences of the UBC domain of the 39 OsUBCs (Fig 3). The tryptophan (W) located at the C-terminal side of the active cysteine was conserved in all 39 OsUBCs, and ubiquitin thioester intermediate interaction residues (Ub1 to Ub5) were found to surround the active cysteine site. The consensus active site motif ‘HPN’ was found at seven to eight amino acids from the N-terminal side of the active cysteine, though OsUBC45 lacked the HPN motif. Additionally, the strongly conserved motif (PxxPP) was found at seven to 11 amino acids from the N-terminal side of the HPN motif (Fig 3).

### Expression analysis of *OsUBC* genes in various organs at different developmental stages

To analyze the expression patterns of the *OsUBC* genes in different tissues and organs in rice, we examined publically available transcriptomics resources including EST tags, microarray data, and MPSS tags, as described in detail previously [28].

The initial phase of our EST profile analysis searched the NCBI UniGene database (http://www.ncbi.nlm.nih.gov/unigene/) for the corresponding full-length cDNA (FL-cDNA) and ⁄ or ESTs of the 39 putative *OsUBC* genes. 36 of 39 (92.31%) *OsUBC* genes had both FL-cDNA and EST evidence (Table 1; S1 Table), indicating that most of the putative genes we examined were actually expressed in rice. Various *OsUBC* genes showed high expression levels in stems, roots, leaves, panicles, and seeds, and several of these genes had tissue-specific expression patterns: *OsUBC46* in callus; *OsUBC8/23* in flowers; *OsUBC16* in leaves; *OsUBC7/33* in panicles; *OsUBC1/2/45* in roots; *OsUBC11/13* in stems; and *OsUBC12/27/44/47* in the shoot apical meristem (SAM) (S1 Table).

**Fig 4:**
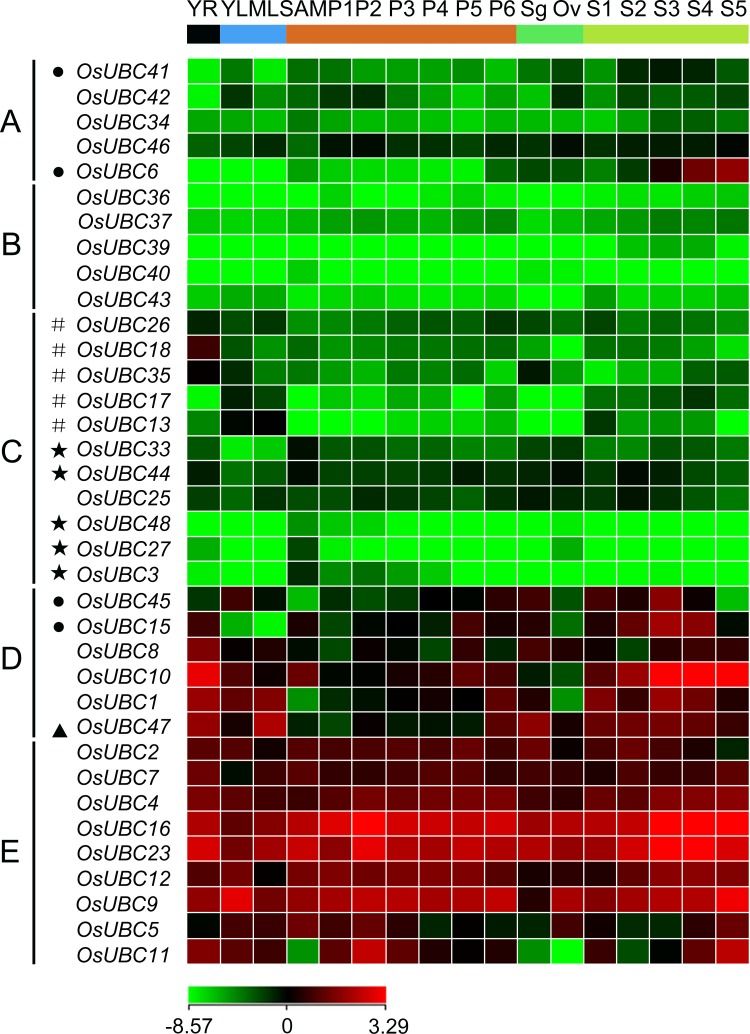
Expression profiles of *OsUBC* genes in various organs. The microarray data sets (GSE6893 and 7951) of *OsUBC* genes expression in organs at various developmental stages were reanalyzed. A heat map representing hierarchical cluster in various organs was generated. Color key represents average log2 expression values of *OsUBC* genes. The colour scale (representing average log signal values) is shown at the bottom. Samples are mentioned at the top of each lane: YR, roots from 7-day-old seedlings; ML, mature leaf; YL, leaves from 7-day-old seedling; SAM, shoot apical meristem; different stages of panicle development: P1, 0–3 cm; P2, 3–5 cm; P3, 5–10 cm; P4, 10–15 cm; P5, 15–22 cm; P6, 22–30 cm; Sg, stigma; Ov, ovule; different stages of seed development: S1, 0–2 dap (day after pollination); S2, 3–4 dap; S3, 5–10 dap; S4, 11–20 dap; S5, 21–29 dap. Genes that share similar expression patterns are divided into five groups: (A) preferential expression during seed development; (B) low expression in all examined organs; (C) high expression in specific organs; (D) high expression levels in particular vegetative and reproductive organs; (E) high expression levels in all examined organs. Asterisks, hash symbols, triangles and rounds indicate the genes with preferential expression level in SAM, YR and/or ML/YL, P1–P6 and S1–S5, respectively.

The expression profiles of *OsUBC* genes were also analyzed by using Microarray data (http://signal.salk.edu/cgi-bin/RiceGE). Data for the following tissues was examined: young roots (YR), mature leaves (ML), young leaves (YL), shoot apical meristem (SAM), panicles (P1-P6), stigmas (Sg), ovules (Ov) and seeds (S1–S5). At least one probe for 36 of 39 *OsUBC* genes was present on the Affymetrix rice whole-genome array platform (GPL2025). A hierarchical cluster display generated from the average log signal values for the 36 *OsUBC* genes in the selected tissues summarized the differential expression patterns of these genes (Fig 4; S2 Table). The expression patterns of these 36 *OsUBC* genes could be divided into five major groups (Fig 4). Group A included 5 genes that showed relatively high expression levels during seed development. For example, *OsUBC6* was highly expressed in S3–S5 and *OsUBC41* was expressed abundantly in S2–S4. Conversely, group B contained 5 genes that showed relatively low expression levels in all of the sample types investigated. Group C comprised 11 genes that showed abundant expression levels in roots, leaves, or SAM: *OsUBC18/26/35* in YR; *OsUBC13/17* in YL and ML; *OsUBC3/27/33/44/48* in SAM. Group D consisted of 6 genes which showed relatively high expression levels in particular vegetative and reproductive organs: *OsUBC45* in YL, Sg, and S1–S4; *OsUBC15* in YR, P5 and S2–S4; *OsUBC10* in YR, SAM, P5 and S1–S5; *OsUBC1* in YR, YL, ML, P6 and S1–S5; *OsUBC47* in YR, ML, P6, Sg, and S1–S4. The nine *OsUBC* genes in group E showed high expression levels in all examined organs, though there were five genes with relatively low expression in particular organs (*OsUBC2* in S5, *OsUBC5* in P4–P6, Sg, and S1–S3, and *OsUBC11* in SAM, Sg, Ov, and S2). Additionally, MPSS data (http://mpss.udel.edu/rice/) analysis showed that the expression of several *OsUBC* genes were highly similar to the results from the microarray data. For example, *OsUBC2/4/7/9/16/23* showed high expression levels in most examined organs, *OsUBC35* in YR, and *OsUBC10* in YR, SAM and immature panicle (S3 Table).

**Fig 5:**
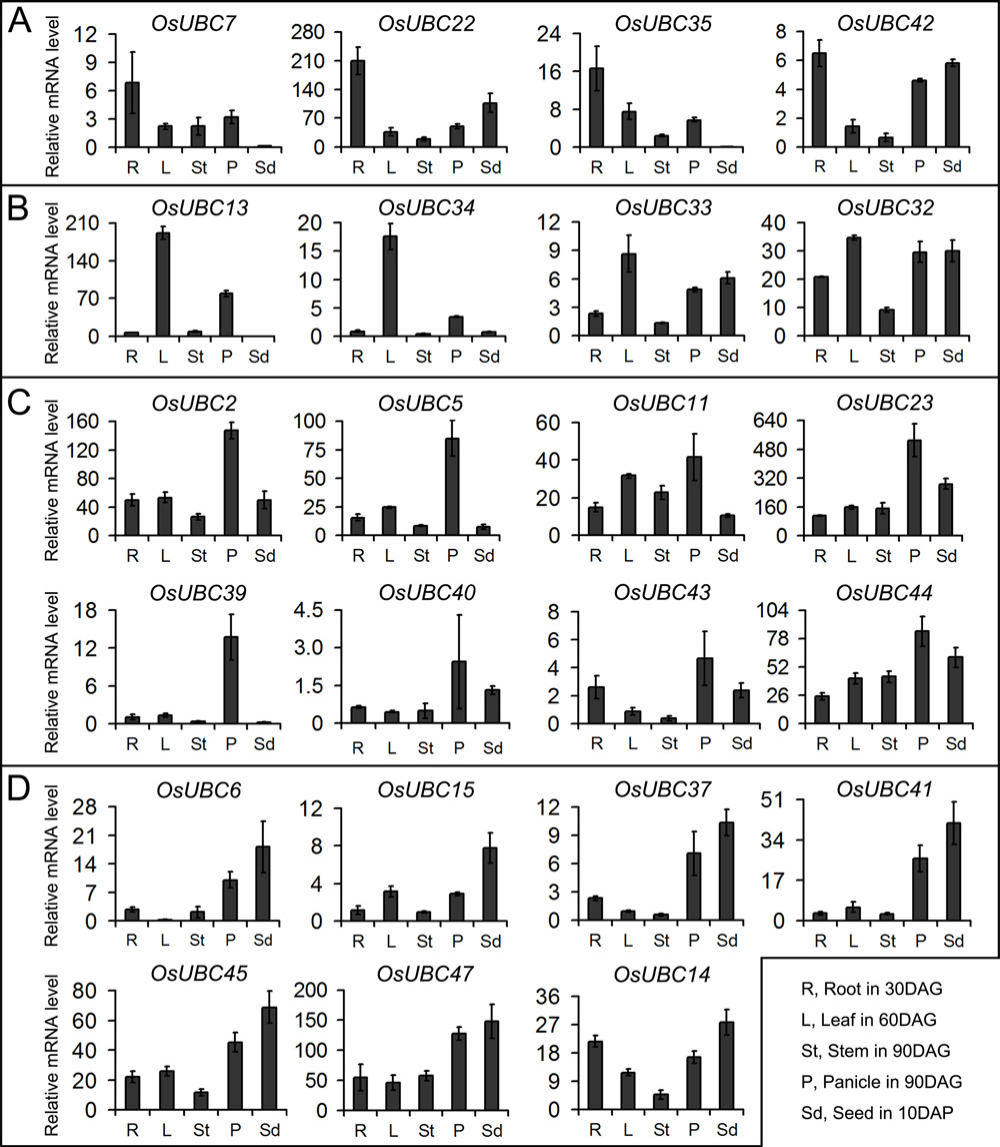
Real-time PCR analysis of tissue-specific expression of the *OsUBC* genes. Relative mRNA levels of individual genes normalized to *UBQ5* are shown. The genes with preferential expression levels in roots (A), leaves (B), panicles (C) and seeds (D) were showed. Error bars indicate standard deviations of independent biological replicates (n = 2 or more).

To validate the results of digital expression analysis, we used real-time PCR analysis to confirm the expression levels of the *OsUBC* genes in rice. The results showed that the real-time PCR results were in general agreement with the microarray and MPSS tag data. For example, *OsUBC26/35* are predominantly expressed in roots, leaves, and panicles (Fig 5A; S4E Fig), and *OsUBC35* is also expressed in stigmas (Fig 4). *OsUBC13* is specifically expressed in leaves, and had extremely low or no expression in roots, stems, and seeds (Fig 5B). *OsUBC46* was predominantly expressed in leaves and panicles (S4B Fig). *OsUBC2/11/23* were abundantly expressed during the processes of panicles (Fig 5C). *OsUBC6/9/14/15/37/41/45/47* were mainly expressed during the processes of seed development (Fig 5D; S4C Fig).

### Regulation of *OsUBC* gene expression in response to abiotic stresses

**Fig 6:**
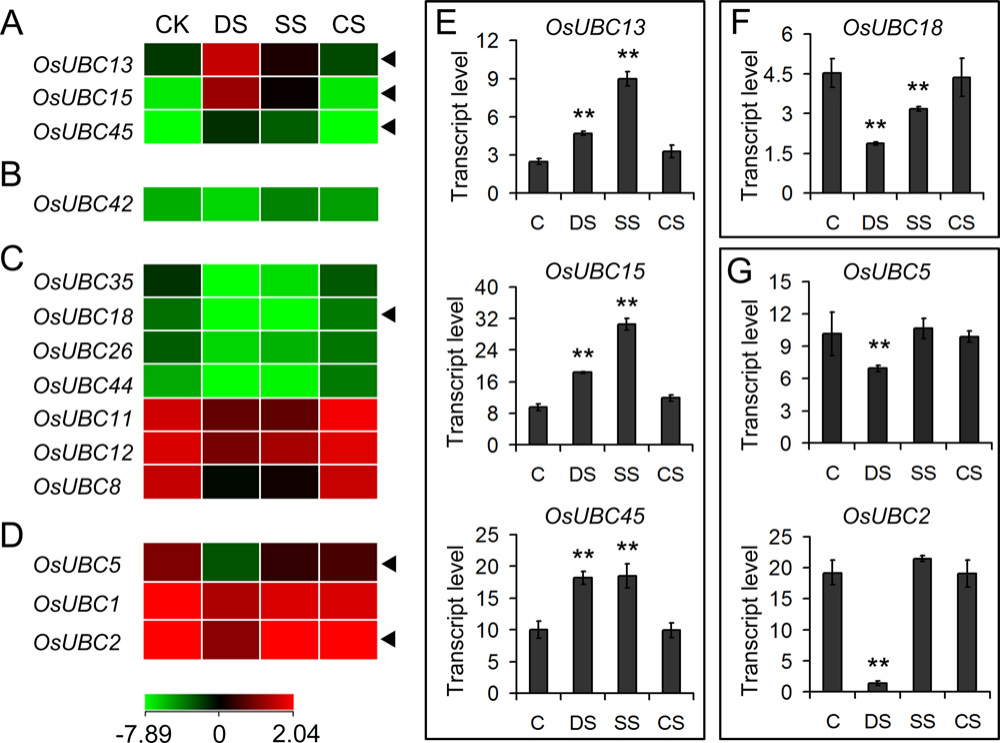
Differential expression profiles of *OsUBC* genes under abiotic stresses. The microarray data sets (GSE6901) of gene expression under various abiotic stresses were used for cluster display. The average log signal values of *OsUBC* genes under control and various stress conditions (indicated at the top of each lane) are presented by a heat map. Only those genes that exhibited >2-fold or more differential expression, under any of the given abiotic stress conditions, are shown. (A) Up-regulated by drought and salt stresses; (B) Up-regulated by salt stress; (C) Down-regulated by drought and salt stresses; (D) Down-regulated by drought stress. The color scale representing average log signal values is shown at the bottom. The representative *OsUBC* genes differentially expressed under different abiotic stresses for which real-time PCR analysis was performed are indicated by black triangle at the right. The results are shown for confirmation of the differential expression of *OsUBC* genes up-regulated by drought and salt stresses (E), down-regulated by drought and salt stresses (F), and down-regulated by drought stress (G). Error bars indicate standard deviations of independent biological replicates (n = 3 or more). Two asterisks (**, P <0.01) represent significant differences between the controls and treatments. C, control; DS, drought stress; SS, salt stress; CS, cold stress.

To examine whether abiotic stress altered the expression of *OsUBC* genes in rice, we investigated microarray (GSE6901) data for 7-day-old seedlings that had been subjected to drought (DS), salt (SS), or cold (CS) treatment. A total of 14 genes were significantly (*P* < 0.05) down- or up-regulated (<0.5 or >2-fold) in at least one of the stress conditions examined, as compared with the control (C) (Fig 6A–6D). The transcriptional levels of three genes (*OsUBC13/15/45*) were significantly up-regulated by both drought and salt stresses (Fig 6A). *OsUBC42* was specifically down- regulated by drought but up-regulated by salt stress (Fig 6B). Seven genes (*OsUBC35/18/26/44/11/12/8*) were significantly down-regulated by both drought and salt stresses (Fig 6C). Three genes (*OsUBC5/1/2*) were down-regulated by drought stress (Fig 6D). Cold stress, however, did not influence the expression any of the *OsUBC* genes (Fig 6A–6D).

We used real-time PCR analysis to test the expression levels of six representative *OsUBC* genes in 7-day-old seedlings treated for 3 h with various stress conditions. We found that the quantitative RT-PCR results were in very good agreement with the microarray data (Fig 6E–6G). The expression levels of *OsUBC13/15/45* were all significantly up-regulated by both drought and salt stresses (Fig 6E). *OsUBC18* expression was down-regulated by both drought and salt stress (Fig 6F). *OsUBC5/2* expression was down-regulated by drought stress (Fig 6G). These results suggest that *OsUBC* genes participate in abiotic stress signaling pathways and may play important roles in plant responses to these stresses in rice.

### Differential expression of *OsUBC* genes in response to hormone treatments

**Fig 7:**
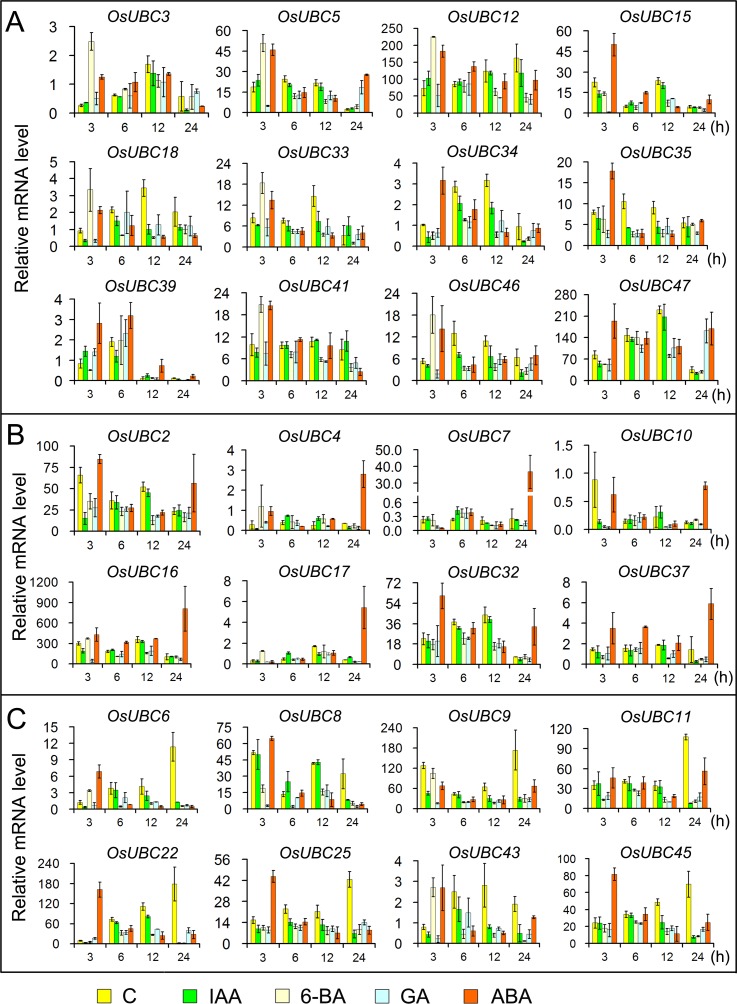
Expression analysis of *OsUBC* genes under different hormone treatments. X-axis indicates time course/treatment and Y-axes are scales of relative expression level. (A) Showing the genes up-regulated by 6-BA or ABA at 3h; (B) Showing the genes up-regulated by ABA at 24h; (C) Showing the genes down-regulated by the four hormones at 12h and 24h; Error bars indicate standard deviations of independent biological replicates (n = 3 or more). C, control; *IAA*, indole-3-acetic acid; *6-BA*, 6-Benzylaminopurine; *GA*, gibberellin acid; *ABA*, abscisic acid; h, hour.

To determine if *OsUBCs* in rice are involved in phytohormone signaling, we investigated expression profiles for *OsUBCs* family under different hormone treatments. Total RNA was isolated from 7-day-old seedlings of *Nipponbare* rice treated with IAA (Indole-3-Acetic Acid), 6-BA (6-Benzylaminopurine), GA (Gibberellin Acid), and ABA (Abscisic Acid), and the expression levels of the *OsUBCs* were evaluated using quantitative RT-PCR. The results showed that the expressions of 12 genes (*OsUBC3/5/12/15/18/33/34/35/39/41/46/47*) were up-regulated under 6-BA or/and ABA and down-regulated under IAA or/and GA at 3h time point (Fig 7A), hinting that these genes might be involved in plant early response to the hormones signaling; *OsUBC2/4/7/10/16/17/32/37* were markedly induced by ABA at 24h (Fig 7B); the expressions of *OsUBC6/8/9/11/22/25/43/45* were induced by 6-BA or/and ABA at 3h, but were reduced by the four hormones at 12h and 24h (Fig 7C), suggesting that they could play a complex role in hormones signaling.

Under IAA treatment, the transcript levels of five genes (*OsUBC18/25/34/35/43*) were continuously decreased throughout the IAA treatment time courses, while four genes (*OsUBC3/11/37/45*) were down-regulated at the later time points of the treatment, their expression levels were evidently decreased by >3-fold (Fig 7). Two genes (*OsUBC39/40*) were induced at the early time points of the treatment, but were reduced at the late time points (Fig 7A; S5 Fig). Nine genes (*OsUBC5/12/15/16/23/27/32/41/47*) were not responsive to IAA. These data reveal that the various *OsUBC* genes have different induction kinetics in response to auxin treatment (Fig 7A; S5 Fig).

Ten genes (*OsUBC4/5/6/12/17/18/33/41/43/46*) were induced at the early time points of the 6-BA treatment, but were reduced at the later time points (Fig 7). The expression levels of five genes (*OsUBC2/8/22/26/34*) were continuously decreased throughout the time course (Fig 7; S5 Fig). Our results indicated that most of the *OsUBC* genes were responsive to 6-BA.

In the GA treatment, the expression levels of five genes (*OsUBC5/14/27/36/47*) showed different changes at the different time points of the treatment. For example, *OsUBC5* was reduced at 3h after GA treatment but increased at 24h time point (Fig 7A). Meanwhile, *OsUBC27*/36 were up-regulated by GA treatment at 3h, but were down-regulated at 12h or 24h (S5 Fig).

For the ABA treatment, 14 genes (*OsUBC3/6/12/15/18/22/25/33/34/35/41/43/45/46*) were induced at the early time points of the treatment, but reduced at the later time points (Fig 7). *OsUBC7/10/17*, however, were dramatically decreased at the early time points but increased at the late time points. It merits mention that *OsUBC7* was increased by >50-fold at the 24h time point of the ABA time series (Fig 7B).

Comparison of the induction kinetics of *OsUBC* genes following treatment with different hormones revealed that the expression levels of most genes decreased under at least two hormones treatments. Five genes (*OsUBC1/9/11/14/26*) were down-regulated by most of the hormone treatments. Moreover, most of the genes reduced by 6-BA were also reduced by GA. Several genes were up-regulated under IAA, 6-BA, GA and ABA treatments. For example, *OsUBC4* was induced by IAA, 6-BA, and ABA treatments (Fig 7B). In addition, *OsUBC5* showed differential response under 6-BA, GA, or/and ABA treatments at different time points, but it is not responsive to IAA (Fig 7A). The hormone- responsiveness spectrum of this family suggested that almost all the genes were responsive to at least two of the four hormones tested in these experiments.

### Comparative expression analysis of *UBC* genes in rice and *Arabidopsis*

To investigate valuable clues for the study of gene function, a comparative analysis of the expression patterns of the rice and *Arabidopsis UBC* genes was performed by using microarray and MPSS data, including roots (R), leaves (L), inflorescences (I), pollens (P), and seeds/siliques (S), as well as plants treated with drought (DS), salt (SS), cold (CS) stresses (S4 Table). Most rice and *Arabidopsis UBC* genes were expressed in at least one data set; only one gene (*AtUBC24*) was not detected in either the microarray or the MPSS data.

**Fig 8:**
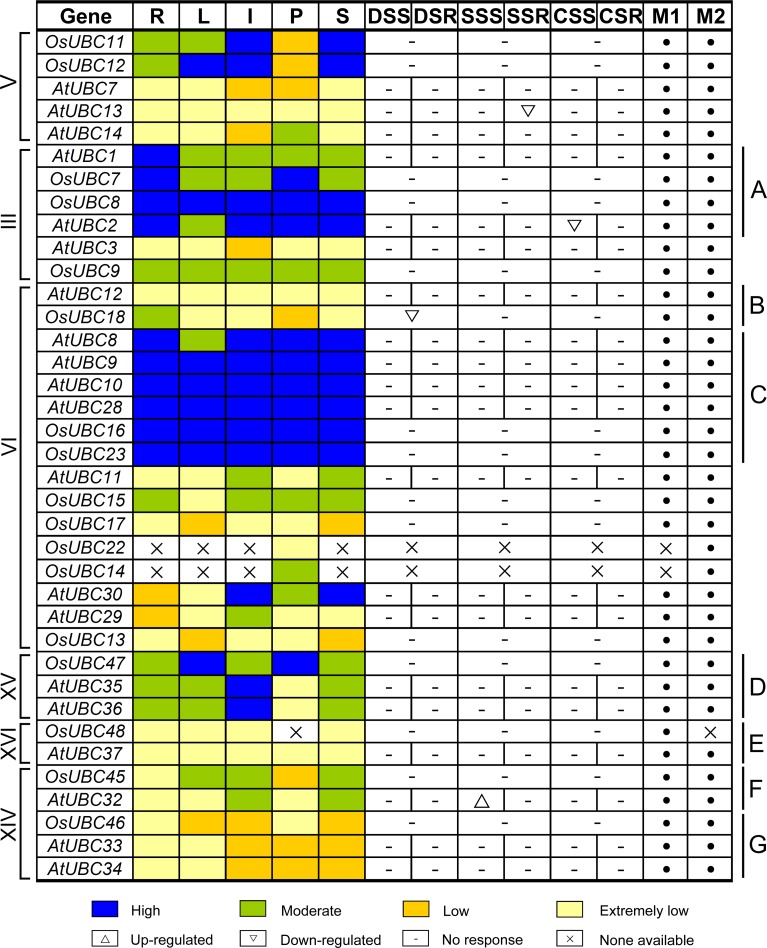
Expression comparison between rice and *Arabidopsis UBC* genes in different organs and under abiotic stresses. The *OsUBC* and *AtUBC* genes in subfamilies V, III, VI, XV, XVI and XIV are displayed according to the order in the corresponding phylogenetic tree (S2 Fig). The expression data of *OsUBC* genes in different organs are combined from microarray and MPSS tags represented by M1 and M2, respectively. The expression data in pollens of *OsUBC* genes were extracted from MPSS tags. The ratios of the absolute values divided by the average of all microarray values were used for analysis (S4 Table). Blue, green, yellow and light yellow boxes indicate high (more than 2 or more than 300 tpm), moderate (between 1 and 2 or between 50 and 300 tpm), low (between 0.5 and 1 or the signature numbers between 0 and 50 tpm), and extremely low (less than 0.5 or no signature is found) expression levels, respectively. R, root; L, leaf; I, inflorescence; P, pollen; S, silique or seed; DSS and DSR; drought stressed shoot and root; SSS and SSR, salt stressed shoot and root; CSS and CSR, cold stressed shoot and root.

After integrating the data from microarray and MPSS tags, we found that several genes with close evolutionary relationships showed similar expression patterns in both species. For example, two genes (*AtUBC1* and *OsUBC7*) in subfamily III were highly expressed in roots and were moderately expressed in leaves, inflorescences, and seeds. *OsUBC8* and *AtUBC2* were highly expressed in all organs examined, though *AtUBC2* was only moderately expressed in leaf (Fig 8A). In subfamily VI, the expression levels of *AtUBC12* and *OsUBC18* were low or extremely low in most organs (Fig 8B); six members (*AtUBC8/9/10/28*, *OsUBC16/23*) were highly expressed in all organs examined, except *AtUBC8* that was moderately expressed in leaves (Fig 8C). Three genes (*OsUBC47*, *AtUBC35/36*) in subfamily XV were moderately or highly expressed in roots, leaves, inflorescences and seeds (Fig 8D). *AtUBC37* and *OsUBC48* in subfamily XVI were expressed at extremely low levels in all tissues examined (Fig 8E). In subfamily XIV, *AtUBC32* and *OsUBC45* were expressed at extremely low levels in roots and pollen, and were moderately expressed in inflorescences and seeds (Fig 8F); Three genes (*OsUBC46*, *AtUBC33/34*) were expressed at low or extremely low levels in all tissues examined (Fig 8G). Similarly, the expression levels of the 13 members in subfamily XI were low or extremely low in most organs (S6 Fig; S6 Table).

In some cases, the expression of *OsUBC* genes differed from their *Arabidopsis* homologs. For example, in subfamily V, *OsUBC11*/*12* from rice were highly or moderate expressed in roots, leaves, inflorescences and seeds, while *AtUBC7/13*/*14* from *Arabidopsis* were expressed at low or extremely low levels in the organs (Fig 8).

**Fig 9:**
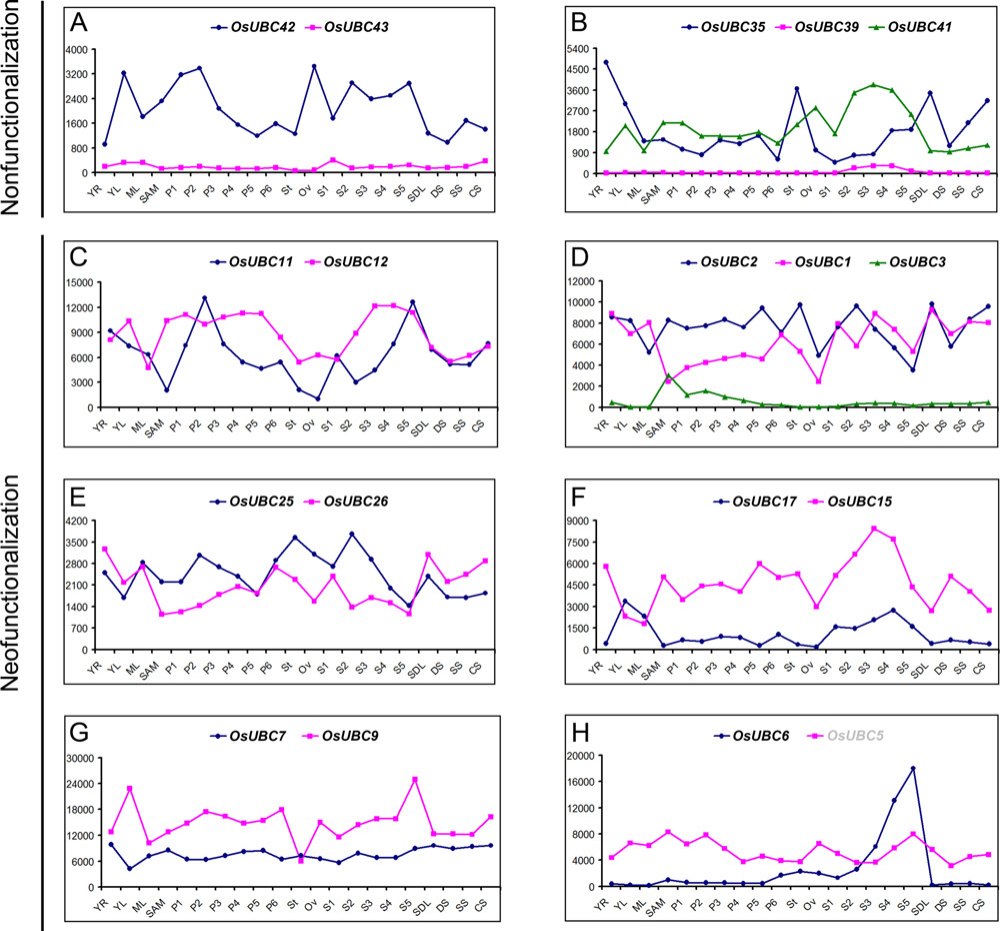
Expression divergence of paralogous *OsUBC* genes involved in duplication. The absolute values of duplicated genes obtained from microarray data were compared in various organs and under abiotic stresses. (A) and (B) Showing gene pairs described as non-functionalization; (C) to (H) Showing gene pairs described as neo-functionalization.

It was interesting to note that 8 duplicated gene groups in rice showed differential expression patterns (Fig 9). One copy of the paralog (*OsUBC42*/*43*, *OsUBC35*/*39*/*41*) had almost negligible expression in all organs (Fig 9A and 9B). For the six groups of paralogous genes (*OsUBC11*/*12*, *OsUBC1/2/3*, *OsUBC25/26*, *OsUBC15/17*, *OsUBC7/9*, and *OsUBC5/6*), the expression patterns were very divergent for one or more of the tissues examined, indicating probable neo-functionalization (Fig 9C–9H). These data reveal that *OsUBC* genes in rice have undergone functional divergence in the due course of evolution. Our evaluation of the expression patterns of these genes provides a foundation for future functional studies of *UBC* genes in both rice and *Arabidopsis*.

## Discussion

### Characterization of rice *UBC* family genes

Ubiquitin-conjugating enzymes are critically important in many aspects of plant growth and development. Considering the potential functional significance of UBC members and the fact that only a few UBC family members have been described in higher plants, it was timely and quite relevant for us to characterize the *UBC* gene family in rice.

In this study, by exhaustive, genome-wide exploration using various databases and tools, a total of 39 ubiquitin-conjugating enzyme encoding genes in the rice genome were identified. This was similar to the number known in the *Arabidopsis* genome (37 members) [13]. Though 48 E2 UBCs in rice were recently identified by Bae et al., 9 of 48 proteins had a UBC domain without an active site Cys residue and could be considered ubiquitin E2 variants (UEVs) [10], including the OsUBC19/20/21/24/28/29/30/31/38. The nine proteins were excluded from further analysis in our study due to lack of the active Cysteine residue. Based on amino acid sequence similarity and phylogenetic analysis, the rice UBC family could be differentiated into fifteen groups, and there were obvious difference in numbers among subfamilies. Subfamily XI had nine members, and subfamily VI had eight members. This is the same as in *Arabidopsis* subfamilies VI. Phylogenetic analysis indicated that the rice *UBC* subfamilies were very much in line with that in *Arabidopsis* (S2 Fig), which revealed that the *UBC* family in rice and *Arabidopsis* formed before the split of monocots and eudicots. In comparison with *Arabidopsis*, the rice genome was found to have fewer *UBC* genes in subfamilies IV, V, VII, VIII, and XV, and lacked subfamily XIII entirely. The change in the number of *OsUBC* members suggested that the *OsUBC* family had undergone functional divergence during the course of evolution.

The UBC proteins were divided into four types, namely class I-IV, and the presence of the N- and/or C- terminal extensions related to their different function [36–37]. Among OsUBC proteins, OsUBC48 (556aa) that belonged into the class III and was missing the N-terminal extension was significantly longer than OsUBC37/39/40/41/42/43 that was ranging from 373–509aa in class IV containing both extensions. Moreover, the primary structure of these class IV members differed considerably compared to OsUBC34 (1063aa), OsUBC35 (876aa) and OsUBC36 (1045aa) that also belonged in class IV (Fig 2; S3 Fig). These divergent structural properties may be responsible for their interactions with E1, E3 or substrates.

### Duplication contributed to the expansion and functional divergence of the *OsUBC* gene family

Gene duplication may occur through three major pathways: chromosomal segmental duplication, tandem duplication, and retroposition, which are thought to be an important means of gene family expansion and functional diversity during evolution [38–39]. In *OsUBCs* family, 15 of 39 (38.5%) *OsUBCs* resulted from segmental duplication, and four genes (10.2%) from tandem duplication, indicating that gene duplication events, particularly segmental duplication, contributed to the expansion of *OsUBCs* family in rice. Interestingly, the tandemly duplicated pairs of genes OsUBC22/23 and OsUBC41/35 are unique within the rice genome compared to other monocots.

It was well known that when gene duplication occur a great divergence in expression dynamics and/or gene function can develop between the two duplicated genes as a result of intense selection pressure [40]. Our comparative analysis of the expression patterns of paralogous *OsUBC* genes indicated that most duplicated *OsUBC* genes exhibited significantly different expression patterns and responded to various abiotic stresses. In the genes of two groups (*OsUBC42/43*, *OsUBC35/39/41*) that resulted from segmental and tandem duplication, one copy of the paralog had negligible expression in all organs and thus seemed to lose its function in the course of evolution, which could be described as nonfunctionalization (Fig 9A and 9B). The fate of the six pairs in Figs 9C–9H could be described as neo-functionalization, as the expression patterns of these paralogous genes were very divergent in one or more of the tissues examined. Additionally, as tandemly duplicated genes, the polypeptides of *OsUBC22* and *OsUBC23* had the same length of 148aa, while the size of OsUBC35 (876aa) and OsUBC41 (509aa) remarkably differed by 367aa (Table 1). These results showed that gene duplication events not only facilitated the expansion of the *OsUBC* family, it also led to the divergence of expression between duplicated genes thus further contributing to the establishment of novel gene functions

### Expression pattern divergence and functional diversity among the *OsUBC* gene family members

The analysis of the temporal and spatial expression patterns of *OsUBC* genes may provide useful information for establishing their putative functions [41]. Our expression analysis of Microarray, MPSS, and EST data indicated that the expression patterns of 36 *OsUBC* genes could be divided into five major groups (Fig 4; S1–S3 Tables). Further, we identified tissue-specific expression of *OsUBC* genes. Of the 36 genes analyzed in this study, only five (*OsUBC13/17/18/*26/*35*) were found to exhibit either predominant or tissue-specific expression in YR, YL or ML, and also five (*OsUBC3/27/33/44/48*) in SAM. In addition, seven genes were preferentially expressed during different development stages of panicles and seeds, and nine genes were most highly expressed in most specific tissues examined (Fig 4D and 4E; S3 Table).

In *Arabidopsis*, *AtUBC1* and *AtUBC2* were ubiquitously expressed in roots, leaves, flowers, and seedlings, and the double mutant *atubc1-1atubc2-1* showed a dramatically reduced number of rosette leaves and an early-flowering phenotype [18]. Our data showed that *OsUBC7* and *OsUBC8*, orthologs of *AtUBC1* and *AtUBC2*, respectively, had high expression levels in most organs, especially in YR, ML, and P2–P5 (Fig 4). Similar expression patterns suggested that these genes might play important roles in leaf formation and panicle development. The real-time PCR expression analysis showed that four genes (*OsUBC13/32/33/34*) were highly expressed in leaves (Fig 5B), indicating that they might play important roles in leaf development in rice. Five genes (*OsUBC7/22/35/36/42*) with predominant expression levels in roots might participate in root system formation (Fig 5A; S4 Fig). Interestingly, five genes (*OsUBC6/9/41/45/47*) exhibited predominant expression levels during the development of both panicles and seeds, which suggested that these genes might be involved in the regulation of reproductive development in rice (Fig 5D; S4 Fig).

### Potential involvement of *OsUBCs* in the regulation of abiotic stress and hormone responses

The expression profile of a gene can provide a valuable clue for studying its function [42]. Our expression analysis using microarray and real-time PCR data revealed that a subset of *OsUBC* genes from different subfamilies showed differential expression patterns under three abiotic stresses (Figs 6 and 8). It is known that E2s have roles in plant responses to UV radiation, high temperature, high salt, and drought [14, 23, 43–44]. *OgUBC1* from wild rice is involved in cellular responses against biotic and abiotic stresses [23]. *AtUBC32* were strongly induced by salt stress in *Arabidopsis* and played a role in the brassinosteroid (BR)-mediated salt tolerance in plant. [22]. The expression of *GmUBC2* was up-regulated by both drought and salt stress in soybean, and *Arabidopsis* plants over-expressing *GmUBC2* were more tolerant to salinity and drought stresses than control plants [45]; the same tolerance was observed for *AhUBC2* in peanut [43], *CmUBC* in *Cucumis melo* [46], and *VrUBC1* in mung bean [26]. Similarly, in our investigation, three genes (*OsUBC13/15/45*) in the *OsUBC* family were significantly up-regulated under salt and drought stresses (Fig 6). On the contrary, the expression analysis from microarray data showed that five genes (*AtUBC13/17/20/26/31*) in *Arabidopsis* were dramatically down-regulated by drought and/or salt stresses (Fig 8; S5 Fig). The expression of several genes (e.g. *OsUBC2/5/18*) was also strongly down-regulated by drought and/or salt stress in rice (Figs 6 and 8). It was notable that *UBCs* from both *Arabidopsis* and rice were not responsive to cold stress. These data indicated that these *OsUBC* genes may play critical roles in drought and salt stresses signaling in rice.

Hormone-signaling is integral in plant responses to abiotic stresses, and *UBC* genes may play roles in hormone-mediated stresses responses [5–7]. It has been reported that a network of rice genes is associated with environmental factors and developmental cues [47]. Using expression analysis from microarray data and qRT-PCR, we found that several *OsUBCs* (such as *OsUBC13/15/45*), which were predominantly expressed in leaves, panicles and/or seeds (Figs 4 and 5), were significantly up-regulated by drought and salt stresses (Fig 6), and by ABA (Fig 7; S5 Fig). Three genes (*OsUBC2/5/18*) whose expressions were reduced by drought stress were highly expressed in panicles (Figs 5 and 6; S4 Fig), and were significantly down-regulated after 12h under 6-BA, GA and ABA treatments (Fig 7), indicating that these genes may be involved in plant growth and response to different abiotic stress conditions during reproductive development. These data suggested that a number of *OsUBCs* were likely to be involved in hormone-mediated developmental processes and stress responses.

## Supporting Information

S1 Fig Intron-exon structures of *OsUBC* Genes. Names for the genes are on the left. Introns phases 0, 1 and 2 are indicated by numbers 0, 1 and 2, respectively. The green boxes, exons; black lines, introns; blue boxes, UTR (Un-translated regions).
(TIF)

S2 Fig Phylogenetic analysis of OsUBCs and AtUBCs. The phylogenetic tree of all UBCs from *Arabidopsis* and rice after multiple sequence alignment using the full-length protein sequences is constructed by neighbor-joining method. Scale bar represents 0.1 amino acid substitution per site. The branches of different subfamilies are marked by different colors.
(TIF)

S3 Fig Explanative structure of four OsUBC proteins. The 3D structures of the OsUBC domain were shown from the four types, including class I (A), Class II (B), class III (C), class IV (D). The conserved active-site cysteines (CYS) were shown as green sticks. Conserved secondary structural elements were indicated (α-helices in pink, β-sheets in light blue, and loops in brown). The structures had been adapted by the 3D X-ray structure of UbE2D2 (PDB code E2SK).
(TIF)

S4 Fig Real-time PCR analysis of tissue-specific expression of the *OsUBC* genes. Relative mRNA levels of individual genes normalized to *UBQ5* are shown. The genes with preferential expression levels in roots and panicles (A), leaves and panicles (B), leaves, panicles and seeds (C), roots, panicles and seeds (D), roots, leaves and panicles (E), roots stems and panicles (F) were showed. Error bars indicate standard deviations of independent biological replicates (n = 2 or more).
(TIF)

S5 Fig Expression analysis of *OsUBC* genes under different hormone treatments. X-axis indicates time course/treatment and Y-axes are scales of relative expression level. Error bars indicate standard deviations of independent biological replicates (n = 3 or more). *IAA*, indole-3-acetic acid; *6-BA*, 6-Benzylaminopurine; *GA*, gibberellin acid; *ABA*, abscisic acid. h, hour.
(TIF)

S6 Fig Expression pattern of *OsUBCs* and *AtUBCs* in nine subfamilies. The expression analysis of *OsUBC* and *AtUBC* genes in subfamilies I, VIII, IX, XII, II, IV, X, XIII and XII are displayed according to the order in the S2 Fig. The analysis for expression data of *OsUBC*s and *AtUBCs* in different organs were performed by the same way within Fig 8. R, root; L, leaf; I, inflorescence; P, pollen; S, silique or seed; DSS and DSR; drought stressed shoot and root; SSS and SSR, salt stressed shoot and root; CSS and CSR, cold stressed shoot and root.
(TIF)

S1 Table The EST expression profiles of *OsUBC* genes. (DOC)

S2 Table The microarray analysis of *OsUBC* genes in various organs. (DOC)

S3 Table The MPSS analysis of *OsUBC* genes. (DOC)

S4 Table Data for expession comparison of *OsUBC* and *AtUBC* genes in Fig 8. (DOC)

S5 Table Primers used in qRT-PCR of *OsUBC* genes. (DOC)

S6 Table Data for expession comparison of *OsUBC* and *AtUBC* genes in S6 Fig. (DOC)
