# Supplementary figures and images for: Characterization of the Ubiquitin-Conjugating Enzyme Gene Family in Rice and Evaluation of Expression Profiles under Abiotic Stresses and Hormone Treatments
Source: PLoS One. 2015 Apr 22;10(4):e0122621. doi: 10.1371/journal.pone.0122621 (PMC4406754; doi:10.1371/journal.pone.0122621)

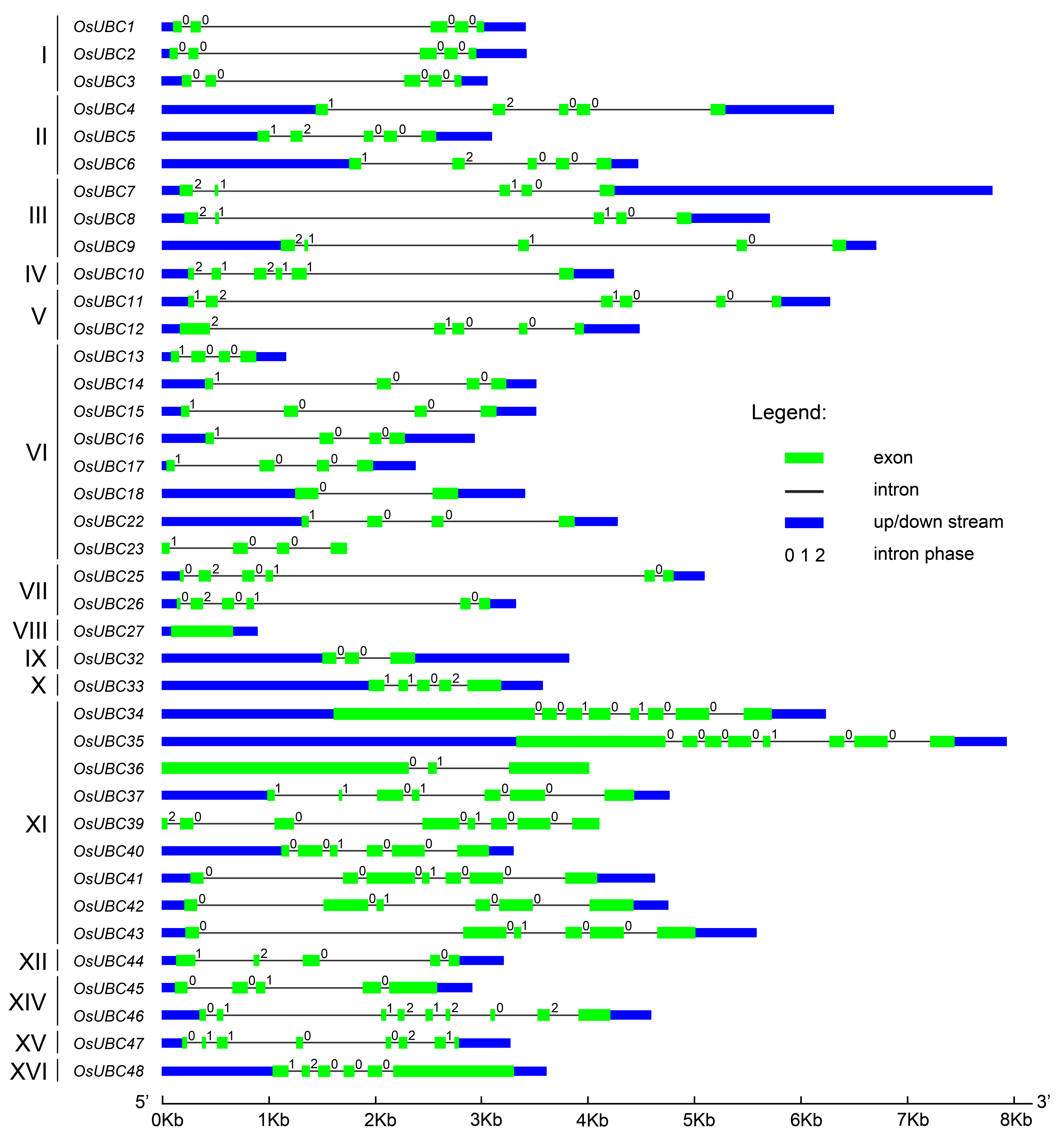

Supplement: S1 Fig — Names for the genes are on the left. Introns phases 0, 1 and 2 are indicated by numbers 0, 1 and 2, respectively. The green boxes, exons; black lines, introns; blue boxes, UTR (Un-translated regions). (TIF) [file pone.0122621.s001.tif]

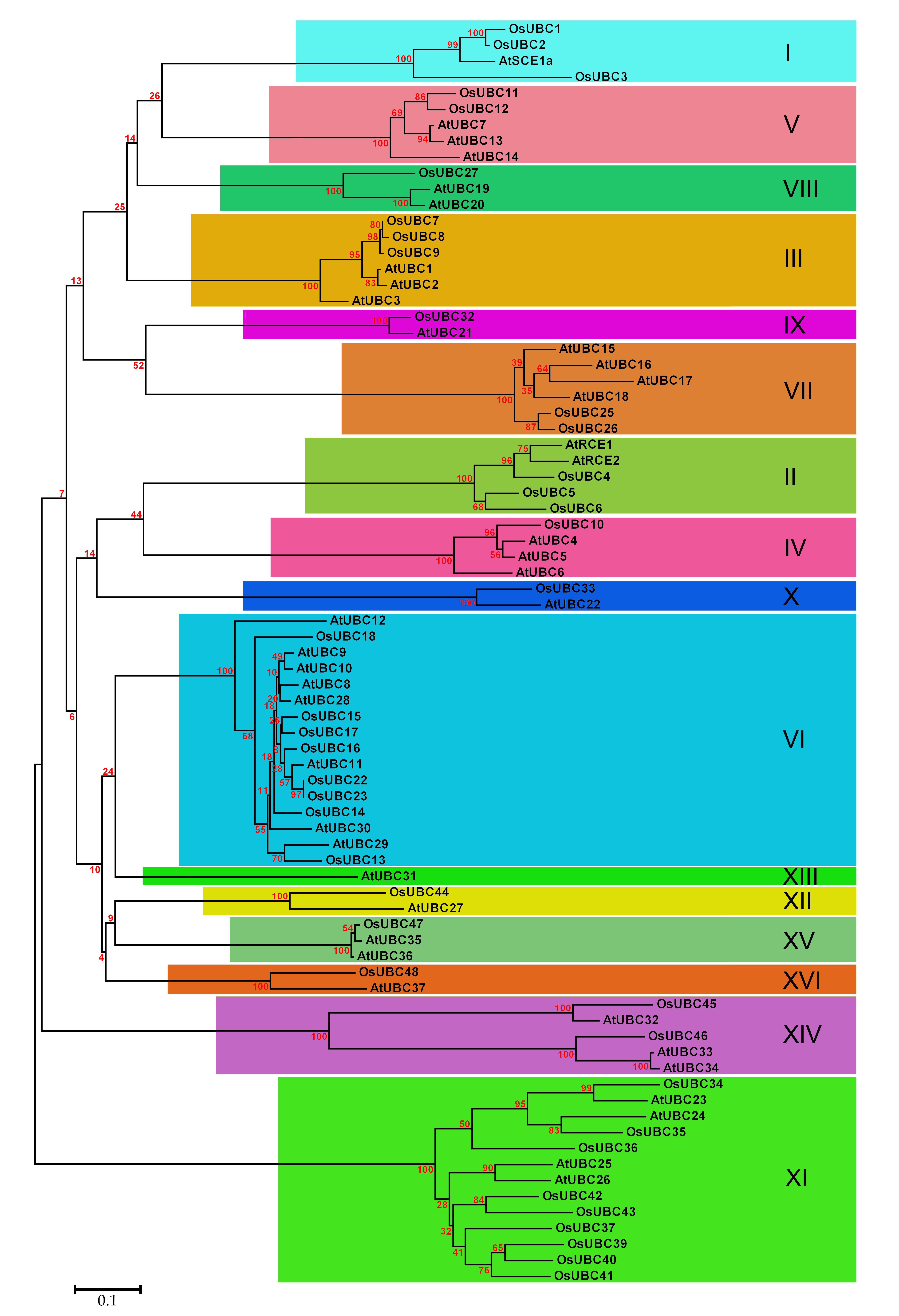

Supplement: S2 Fig — The phylogenetic tree of all UBCs from Arabidopsis and rice after multiple sequence alignment using the full-length protein sequences is constructed by neighbor-joining method. Scale bar represents 0.1 amino acid substitution per site. The branches of different subfamilies are marked by different colors. (TIF) [file pone.0122621.s002.tif]

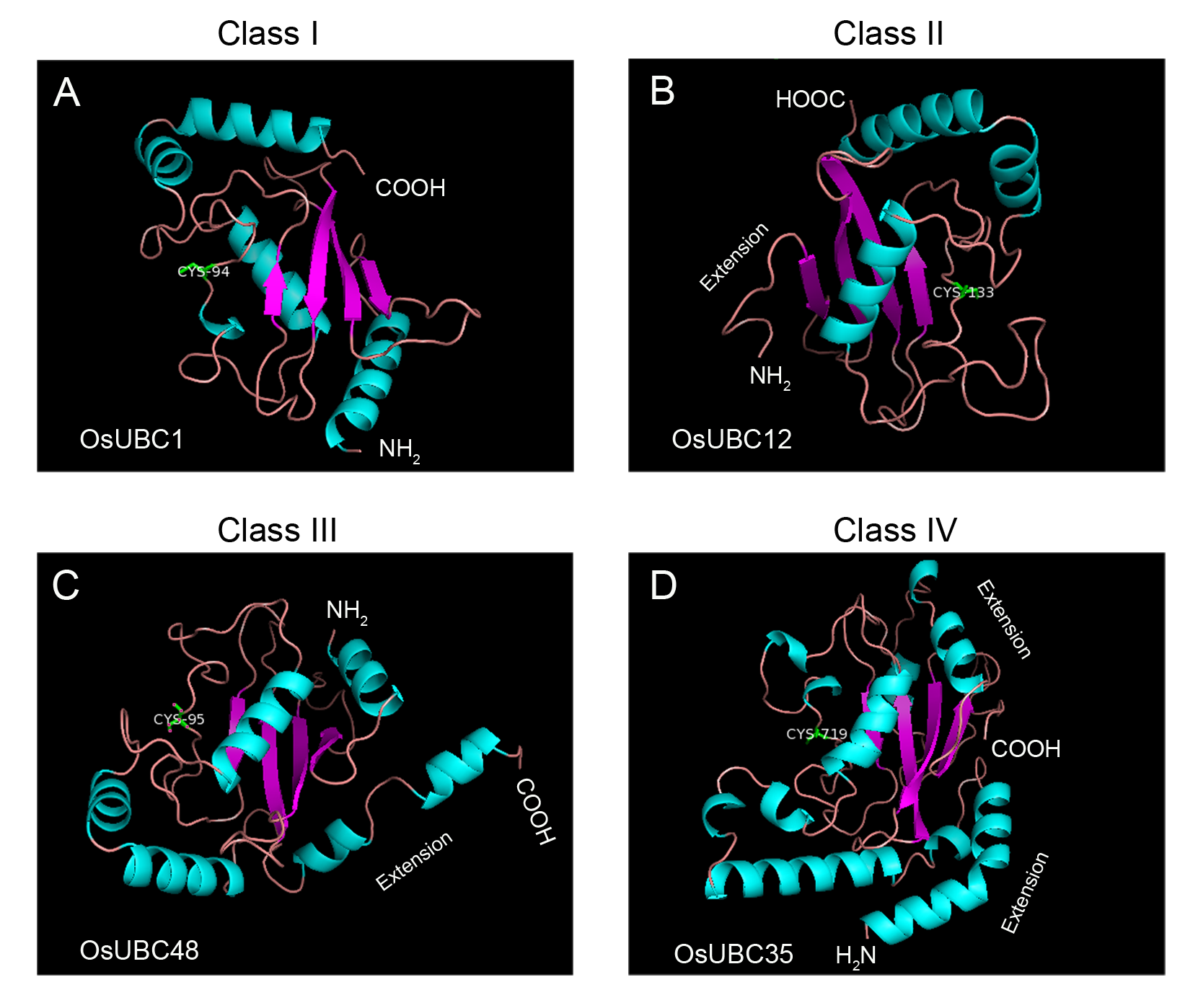

Supplement: S3 Fig — The 3D structures of the OsUBC domain were shown from the four types, including class I (A), Class II (B), class III (C), class IV (D). The conserved active-site cysteines (CYS) were shown as green sticks. Conserved secondary structural elements were indicated (α-helices in pink, β-sheets in light blue, and loops in brown). The structures had been adapted by the 3D X-ray structure of UbE2D2 (PDB code E2SK). (TIF) [file pone.0122621.s003.tif]

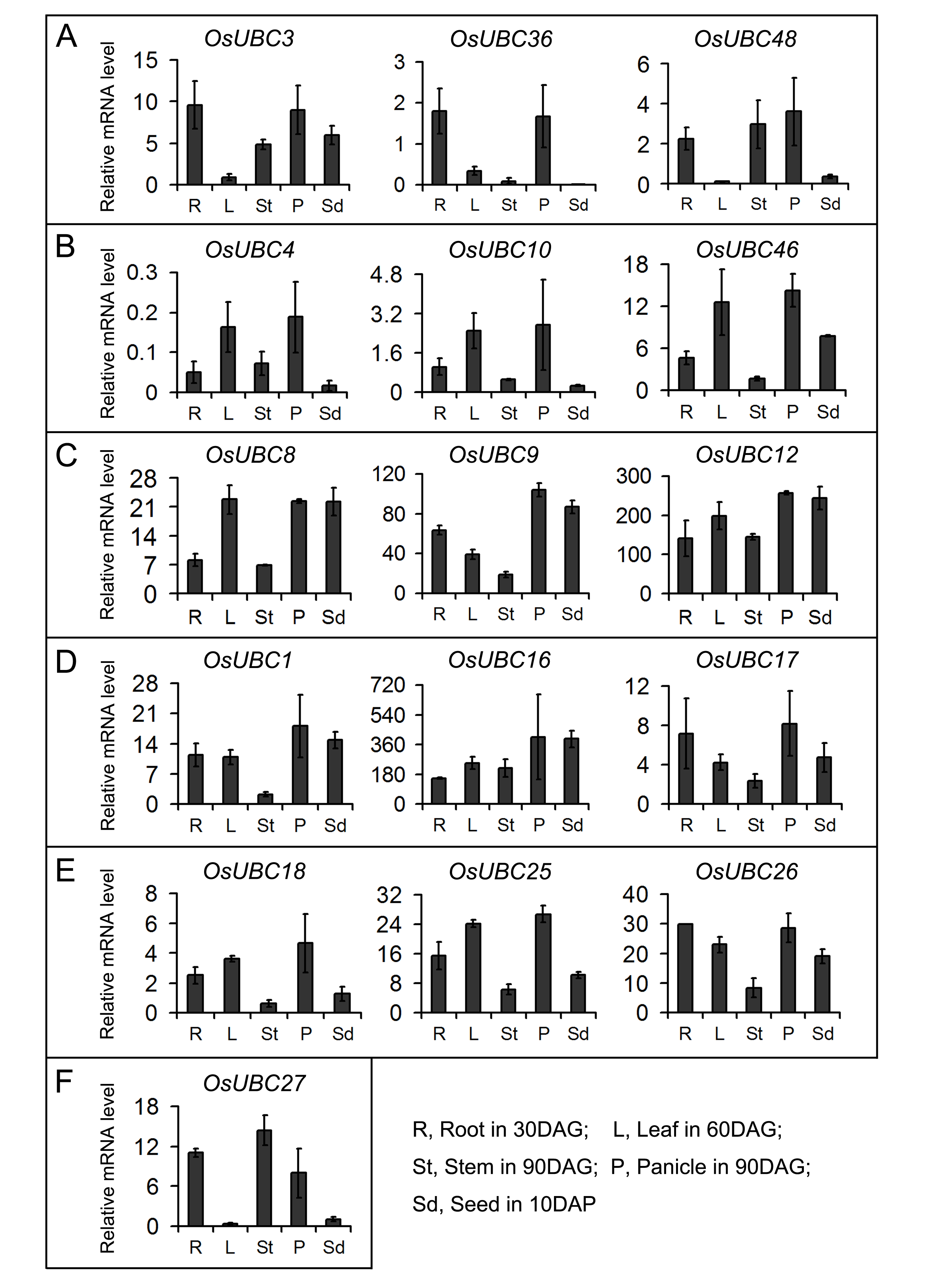

Supplement: S4 Fig — Relative mRNA levels of individual genes normalized to UBQ5 are shown. The genes with preferential expression levels in roots and panicles (A), leaves and panicles (B), leaves, panicles and seeds (C), roots, panicles and seeds (D), roots, leaves and panicles (E), roots stems and panicles (F) were showed. Error bars indicate standard deviations of independent biological replicates (n = 2 or more). (TIF) [file pone.0122621.s004.tif]

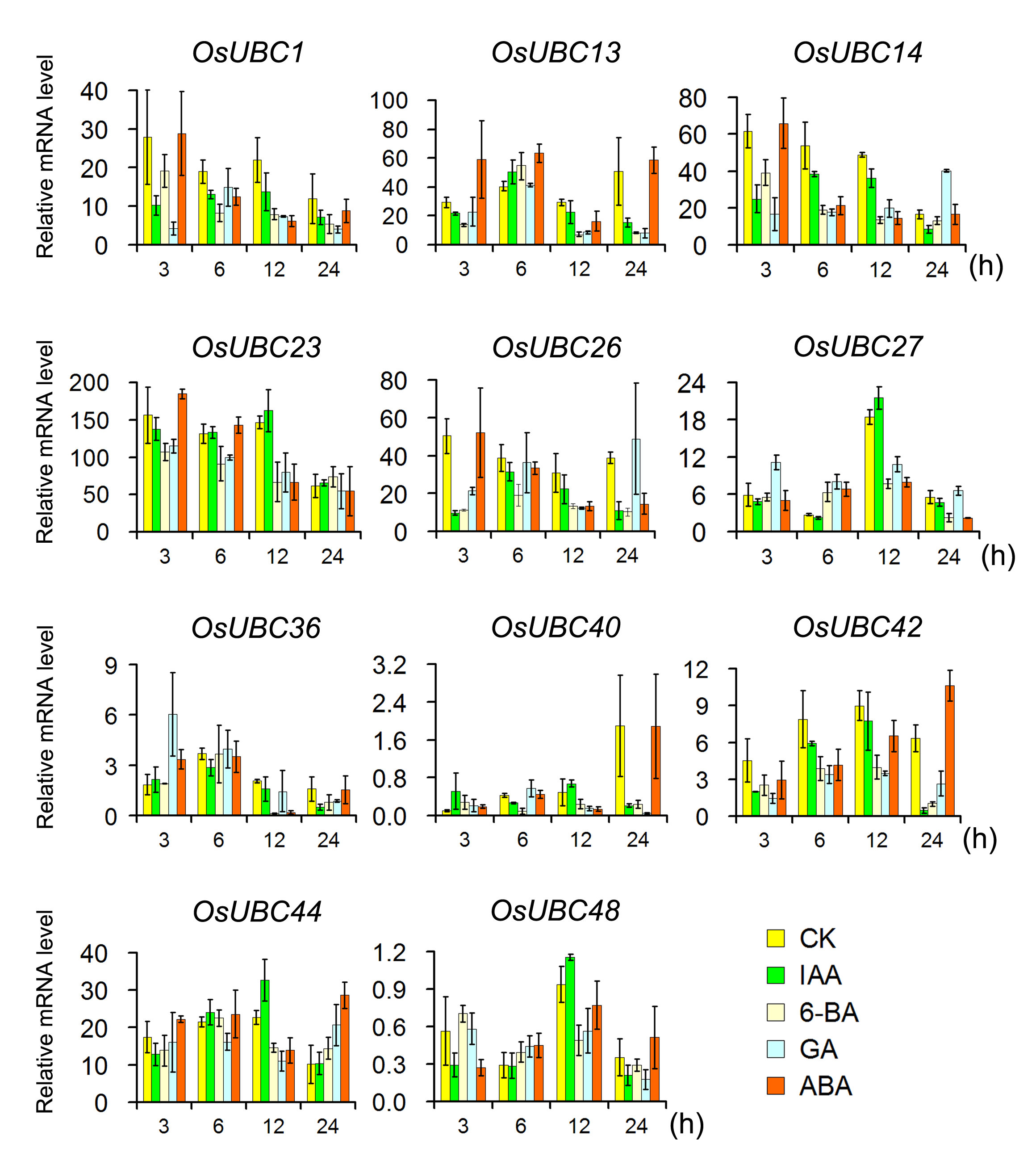

Supplement: S5 Fig — X-axis indicates time course/treatment and Y-axes are scales of relative expression level. Error bars indicate standard deviations of independent biological replicates (n = 3 or more). IAA, indole-3-acetic acid; 6-BA, 6-Benzylaminopurine; GA, gibberellin acid; ABA, abscisic acid. h, hour. (TIF) [file pone.0122621.s005.tif]

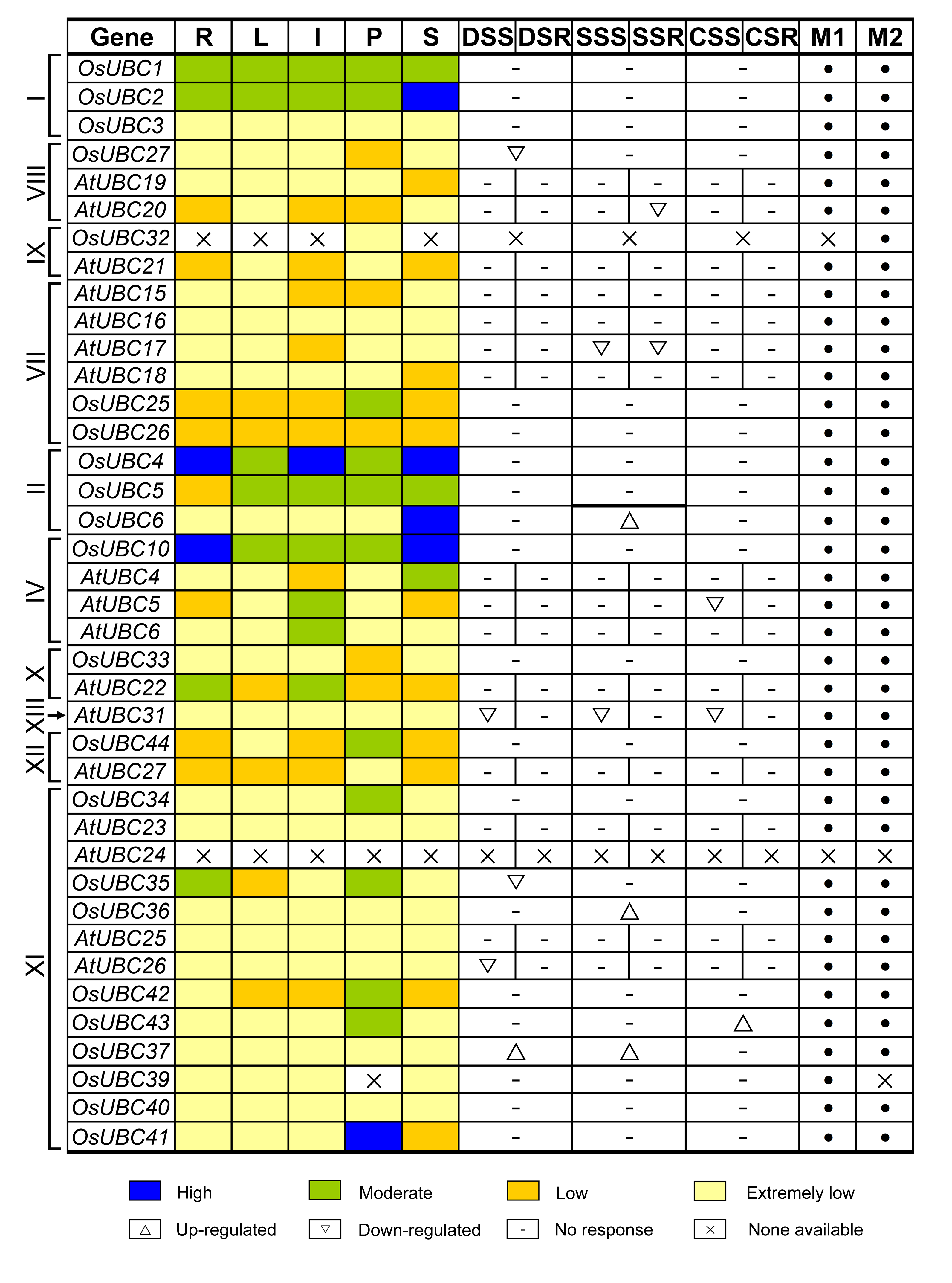

Supplement: S6 Fig — The expression analysis of OsUBC and AtUBC genes in subfamilies I, VIII, IX, XII, II, IV, X, XIII and XII are displayed according to the order in the S2 Fig. The analysis for expression data of OsUBCs and AtUBCs in different organs were performed by the same way within Fig 8. R, root; L, leaf; I, inflorescence; P, pollen; S, silique or seed; DSS and DSR; drought stressed shoot and root; SSS and SSR, salt stressed shoot and root; CSS and CSR, cold stressed shoot and root. (TIF) [file pone.0122621.s006.tif]
