# Supplementary material for: Characterization of the Ubiquitin-Conjugating Enzyme Gene Family in Rice and Evaluation of Expression Profiles under Abiotic Stresses and Hormone Treatments
Source: PLoS One. 2015 Apr 22;10(4):e0122621. doi: 10.1371/journal.pone.0122621 (PMC4406754; doi:10.1371/journal.pone.0122621)
Supplement: S1 Table — (DOC) [file pone.0122621.s007.doc]

**Table S1. The ESTs expression profiles of *OsUBC* genes.**

| **Gene** | **Locus** | **C** | **F** | **L** | **P** | **R** | **S** | **St** | **SAM** | **WP** |
| --- | --- | --- | --- | --- | --- | --- | --- | --- | --- | --- |
| *OsUBC1* | LOC_Os10g39120 | 55 | 74 | 148 | 29 | 238 | 0 | 88 | 0 | + |
| *OsUBC2* | LOC_Os03g03130 | 80 | 89 | 74 | 58 | 208 | 0 | 72 | 0 | + |
| *OsUBC3* | LOC_Os04g49130 | 49 | 0 | 0 | 14 | 44 | 92 | 0 | 0 | + |
| *OsUBC4* | LOC_Os10g11260 | 6 | 215 | 62 | 220 | 208 | 246 | 104 | 0 | + |
| *OsUBC5* | LOC_Os08g28680 | 111 | 29 | 39 | 80 | 44 | 61 | 96 | 0 | + |
| *OsUBC6* | LOC_Os09g15320 | 37 | 37 | 11 | 58 | 0 | 61 | 8 | 0 | + |
| *OsUBC7* | LOC_Os07g07240 | 92 | 163 | 113 | 528 | 312 | 246 | 128 | 440 | + |
| *OsUBC8* | LOC_Os05g08960 | 86 | 476 | 119 | 110 | 267 | 431 | 168 | 440 | + |
| *OsUBC9* | LOC_Os03g57790 | 86 | 37 | 199 | 220 | 386 | 185 | 144 | 220 | + |
| *OsUBC10* | LOC_Os10g31000 | 49 | 89 | 79 | 88 | 133 | 123 | 96 | 0 | + |
| *OsUBC11* | LOC_Os01g62244 | 166 | 14 | 199 | 51 | 74 | 185 | 272 | 0 | + |
| *OsUBC12* | LOC_Os05g38550 | 283 | 148 | 273 | 117 | 282 | 401 | 136 | **1983** | + |
| *OsUBC13* | LOC_Os02g02830 | 0 | 0 | 51 | 14 | 14 | 0 | **136** | 0 | + |
| *OsUBC14* | LOC_Os01g46926 | 92 | 96 | 74 | 66 | 89 | 185 | 120 | 0 | + |
| *OsUBC15* | LOC_Os02g16040 | 185 | 37 | 239 | 44 | 119 | 246 | 64 | 0 | + |
| *OsUBC16* | LOC_Os04g57220 | 777 | 357 | **1007** | 565 | 580 | 647 | 376 | 881 | + |
| *OsUBC17* | LOC_Os06g30970 | 185 | 252 | 51 | 29 | 29 | 215 | 80 | 0 | + |
| *OsUBC18* | LOC_Os09g12230 | 0 | 0 | 17 | 14 | 0 | 0 | 8 | 0 | + |
| *OsUBC22* | LOC_Os01g60360 | - | - | - | - | - | - | - | - | - |
| *OsUBC23* | LOC_Os01g60410 | 123 | 476 | 239 | 190 | 74 | 339 | 144 | 440 | + |
| *OsUBC25* | LOC_Os03g47770 | 61 | 7 | 56 | 22 | 74 | 30 | 32 | 0 | + |
| *OsUBC26* | LOC_Os12g44000 | 30 | 133 | 45 | 110 | 0 | 92 | 16 | 0 | + |
| *OsUBC27* | LOC_Os01g16650 | 12 | 0 | 5 | 22 | 14 | 30 | 0 | **220** | + |
| *OsUBC32* | LOC_Os02g42314 | 117 | 119 | 39 | 51 | 29 | 92 | 104 | 0 | + |
| *OsUBC33* | LOC_Os06g45000 | 43 | 14 | 0 | 110 | 14 | 0 | 48 | 0 | + |
| *OsUBC34* | LOC_Os01g03520 | 43 | 22 | 28 | 14 | 0 | 30 | 16 | 0 | + |
| *OsUBC35* | LOC_Os05g48390 | 49 | 7 | 51 | 58 | 59 | 30 | 40 | 0 | + |
| *OsUBC36* | LOC_Os05g06120 | - | - | - | - | - | - | - | - | - |
| *OsUBC37* | LOC_Os01g13280 | 66 | 44 | 0 | 7 | 0 | 0 | 0 | 0 | + |
| *OsUBC39* | LOC_Os01g48580 | - | - | - | - | - | - | - | - | - |
| *OsUBC40* | LOC_Os09g12310 | 12 | 0 | 0 | 7 | 0 | 0 | 8 | 0 | + |
| *OsUBC41* | LOC_Os05g48380 | 43 | 0 | 0 | 66 | 0 | 30 | 0 | 0 | + |
| *OsUBC42* | LOC_Os01g13170 | 111 | 59 | 28 | 117 | 148 | 61 | 56 | 0 | + |
| *OsUBC43* | LOC_Os05g14300 | 49 | 7 | 5 | 22 | 44 | 0 | 0 | 0 | + |
| *OsUBC44* | LOC_Os01g70140 | 30 | 7 | 17 | 51 | 44 | 92 | 64 | **440** | + |
| *OsUBC45* | LOC_Os03g19500 | 80 | 66 | 34 | 36 | 148 | 0 | 32 | 0 | + |
| *OsUBC46* | LOC_Os06g09330 | 160 | 22 | 91 | 51 | 148 | 61 | 64 | 0 | + |
| *OsUBC47* | LOC_Os01g48280 | 148 | 104 | 74 | 88 | 193 | 154 | 104 | 440 | + |
| *OsUBC48* | LOC_Os01g42040 | 30 | 29 | 22 | 14 | 0 | 0 | 0 | 0 | + |

C, callus; F, flower; L, leaf,; P, panicle; R, root; S, seed; St, stem; SAM, shoot apical meristem; WP, whole plant. Underlined and bold indicated specific expression; underlined indicated abundant expression. “+” and “-” represent “exist” and “not exist”, respectively.
