# Supplementary material for: Characterization of the Ubiquitin-Conjugating Enzyme Gene Family in Rice and Evaluation of Expression Profiles under Abiotic Stresses and Hormone Treatments
Source: PLoS One. 2015 Apr 22;10(4):e0122621. doi: 10.1371/journal.pone.0122621 (PMC4406754; doi:10.1371/journal.pone.0122621)
Supplement: S2 Table — (DOC) [file pone.0122621.s008.doc]

**Table S2. The microarray analysis of *OsUBCs*** in various organs.

| **Gene** | **Locus** | **Organs during developmental stages** | | | | | | | | | | | | | | | | |
| --- | --- | --- | --- | --- | --- | --- | --- | --- | --- | --- | --- | --- | --- | --- | --- | --- | --- | --- |
|  |  | **YR** | **ML** | **YL** | **SAM** | **P1** | **P2** | **P3** | **P4** | **P5** | **P6** | **St** | **Ov** | **S1** | **S2** | **S3** | **S4** | **S5** |
| *OsUBC41* | Os05g48380 | -2.26 | -1.14 | -2.22 | -1.05 | -1.07 | -1.50 | -1.50 | -1.53 | -1.35 | -1.80 | -1.11 | -0.69 | -1.40 | -0.39 | -0.25 | -0.35 | -0.84 |
| *OsUBC42* | Os01g13170 | -2.31 | -0.51 | -1.33 | -0.98 | -0.53 | -0.43 | -1.14 | -1.56 | -1.94 | -1.52 | -1.85 | -0.40 | -1.37 | -0.65 | -0.93 | -0.87 | -0.66 |
| *OsUBC34* | Os01g03520 | -1.58 | -1.59 | -1.79 | -1.15 | -1.56 | -1.77 | -1.70 | -1.82 | -2.00 | -1.72 | -1.76 | -1.76 | -1.92 | -1.49 | -0.90 | -0.89 | -1.12 |
| *OsUBC46* | Os06g09330 | -1.37 | -0.97 | -0.59 | -1.48 | -0.27 | -0.18 | -0.67 | -0.66 | -0.94 | -0.56 | -0.67 | -0.19 | -0.66 | -0.42 | -0.37 | -0.39 | -0.10 |
| *OsUBC6* | Os09g15320 | -3.57 | -4.09 | -5.16 | -2.23 | -2.82 | -3.01 | -3.07 | -3.24 | -3.35 | -1.41 | -1.00 | -1.19 | -1.77 | -0.82 | 0.41 | 1.521 | 1.98 |
| *OsUBC36* | Os05g06120 | -5.77 | -5.99 | -6.41 | -5.75 | -4.68 | -5.95 | -6.51 | -5.17 | -4.61 | -5.31 | -6.49 | -6.74 | -5.34 | -4.98 | -5.12 | -4.60 | -4.41 |
| *OsUBC37* | Os01g13280 | -4.49 | -4.71 | -4.72 | -4.11 | -3.56 | -3.34 | -3.79 | -4.00 | -3.42 | -2.95 | -4.83 | -4.17 | -3.72 | -3.39 | -2.74 | -2.94 | -2.60 |
| *OsUBC39* | Os01g48580 | -7.28 | -6.43 | -6.45 | -6.80 | -7.14 | -7.31 | -7.47 | -7.34 | -7.44 | -7.86 | -7.18 | -8.08 | -7.79 | -4.31 | -3.81 | -3.83 | -5.39 |
| *OsUBC40* | Os09g12310 | -6.88 | -6.00 | -6.06 | -4.56 | -6.35 | -6.44 | -6.91 | -6.57 | -7.55 | -5.93 | -5.12 | -5.42 | -7.06 | -5.92 | -5.94 | -5.80 | -6.05 |
| *OsUBC43* | Os05g14300 | -4.5 | -3.83 | -3.81 | -5.21 | -4.77 | -4.55 | -4.99 | -5.22 | -5.06 | -4.81 | -5.98 | -5.81 | -3.48 | -4.90 | -4.61 | -4.60 | -4.20 |
| *OsUBC26* | Os12g44000 | -0.49 | -1.06 | -0.76 | -1.98 | -1.89 | -1.67 | -1.35 | -1.16 | -1.31 | -0.77 | -1.00 | -1.53 | -0.94 | -1.73 | -1.43 | -1.58 | -1.98 |
| *OsUBC18* | Os09g12230 | 0.84 | -1.14 | -2.01 | -1.46 | -2.05 | -1.87 | -1.67 | -1.62 | -1.54 | -1.46 | -2.26 | -4.56 | -1.55 | -1.58 | -1.69 | -2.23 | -3.07 |
| *OsUBC35* | Os05g48390 | 0.07 | -0.60 | -1.72 | -1.64 | -2.13 | -2.50 | -1.67 | -1.82 | -1.48 | -2.90 | -0.32 | -2.18 | -3.28 | -2.54 | -2.46 | -1.31 | -1.26 |
| *OsUBC17* | Os06g30970 | -3.41 | -0.43 | -0.97 | -4.04 | -2.76 | -3.06 | -2.33 | -2.43 | -4.05 | -2.11 | -3.74 | -4.59 | -1.51 | -1.63 | -1.13 | -0.74 | -1.50 |
| *OsUBC13* | Os02g02830 | -1.84 | -0.08 | -0.02 | -5.17 | -4.02 | -3.97 | -3.02 | -2.85 | -2.98 | -2.51 | -6.00 | -5.47 | -0.75 | -2.22 | -2.03 | -2.12 | -4.47 |
| *OsUBC33* | Os06g45000 | -1.18 | -3.25 | -2.79 | -0.22 | -1.22 | -1.11 | -1.47 | -1.38 | -1.82 | -1.81 | -0.96 | -0.79 | -1.73 | -1.87 | -1.11 | -1.22 | -1.67 |
| *OsUBC44* | Os01g70140 | -0.41 | -1.59 | -1.22 | -0.17 | -0.91 | -0.88 | -0.95 | -0.80 | -0.40 | -0.71 | -0.52 | -0.19 | -0.79 | -0.16 | -0.43 | -1.02 | -1.32 |
| *OsUBC25* | Os03g47770 | -0.87 | -1.44 | -0.68 | -1.05 | -1.05 | -0.57 | -0.76 | -0.94 | -1.34 | -0.66 | -0.33 | -0.56 | -0.75 | -0.28 | -0.64 | -1.19 | -1.67 |
| *OsUBC38* | Os01g42040 | -4.37 | -5.40 | -6.19 | -2.00 | -2.75 | -2.92 | -3.43 | -4.24 | -4.59 | -5.10 | -5.43 | -3.27 | -4.14 | -4.43 | -5.34 | -6.36 | -5.94 |
| *OsUBC27* | Os01g16650 | -2.4 | -6.98 | -6.85 | -0.95 | -3.31 | -3.5 | -4.17 | -4.51 | -5.11 | -6.33 | -7.24 | -2.41 | -3.82 | -4.08 | -5.04 | -8.57 | -6.30 |
| *OsUBC3* | Os04g49130 | -3.29 | -6.04 | -6.18 | -0.58 | -1.92 | -1.51 | -2.15 | -2.78 | -4.04 | -4.36 | -6.69 | -6.94 | -5.86 | -3.71 | -3.40 | -3.59 | -4.45 |
| *OsUBC45* | Os03g19500 | -0.29 | 0.35 | -0.09 | -1.03 | -0.25 | -0.43 | -0.32 | -0.00 | -0.02 | 0.25 | 0.35 | -0.46 | 0.38 | 0.18 | 0.75 | 0.07 | -1.05 |
| *OsUBC15* | Os02g16040 | 0.345 | -0.97 | -1.36 | 0.15 | -0.39 | -0.04 | 0.01 | -0.18 | 0.39 | 0.14 | 0.214 | -0.61 | 0.18 | 0.54 | 0.89 | 0.76 | -0.07 |
| *OsUBC8* | Os05g08960 | 0.792 | 0.04 | 0.21 | -0.08 | -0.45 | 0.07 | -0.05 | -0.44 | 0.315 | -0.22 | 0.44 | 0.22 | 0.11 | -0.42 | 0.26 | 0.37 | 0.32 |
| *OsUBC10* | Os10g31000 | 1.47 | 0.50 | 0.09 | 0.66 | -0.05 | -0.02 | 0.158 | 0.24 | 0.60 | 0.44 | -0.18 | -0.49 | 0.53 | 0.98 | 1.64 | 2.25 | 2.35 |
| *OsUBC1* | Os10g39120 | 0.96 | 0.61 | 0.82 | -0.9 | -0.27 | -0.11 | 0.02 | 0.13 | 0.01 | 0.60 | 0.22 | -0.90 | 0.80 | 0.35 | 0.97 | 0.70 | 0.22 |
| *OsUBC47* | Os01g48280 | 0.93 | 0.14 | 1.10 | -0.21 | -0.44 | 0.06 | -0.16 | -0.12 | -0.18 | 0.58 | 0.88 | 0.15 | 0.65 | 0.70 | 0.74 | 0.62 | 0.36 |
| *OsUBC2* | Os03g03130 | 0.91 | 0.85 | 0.19 | 0.86 | 0.721 | 0.77 | 0.87 | 0.74 | 1.05 | 0.65 | 1.09 | 0.12 | 0.73 | 1.08 | 0.70 | 0.31 | -0.37 |
| *OsUBC7* | Os07g07240 | 1.10 | -0.13 | 0.65 | 0.91 | 0.51 | 0.47 | 0.67 | 0.84 | 0.89 | 0.50 | 0.67 | 0.53 | 0.31 | 0.79 | 0.57 | 0.58 | 0.97 |
| *OsUBC4* | Os10g11260 | 1.25 | 0.95 | 0.67 | 0.60 | 0.86 | 1.20 | 1.03 | 1.15 | 1.22 | 1.25 | 0.64 | 0.45 | 1.07 | 0.94 | 1.33 | 1.33 | 1.42 |
| *OsUBC16* | Os04g57220 | 1.79 | 0.97 | 1.35 | 1.86 | 2.28 | 2.85 | 2.16 | 2.09 | 1.99 | 2.15 | 1.61 | 1.83 | 1.85 | 1.98 | 2.70 | 3.27 | 3.29 |
| *OsUBC23* | Os01g60410 | 2.2 | 1.18 | 1.63 | 2.03 | 1.43 | 2.37 | 1.80 | 1.67 | 1.98 | 1.81 | 1.80 | 1.52 | 1.60 | 2.16 | 2.74 | 2.80 | 2.21 |
| *OsUBC12* | Os05g38550 | 0.84 | 1.18 | 0.06 | 1.19 | 1.29 | 1.13 | 1.25 | 1.31 | 1.30 | 0.89 | 0.26 | 0.47 | 0.33 | 0.96 | 1.42 | 1.42 | 1.32 |
| *OsUBC9* | Os03g57790 | 1.48 | 2.33 | 1.15 | 1.49 | 1.69 | 1.93 | 1.85 | 1.69 | 1.77 | 1.98 | 0.39 | 1.72 | 1.35 | 1.66 | 1.80 | 1.80 | 2.45 |
| *OsUBC5* | Os08g28680 | -0.04 | 0.53 | 0.44 | 0.86 | 0.49 | 0.79 | 0.33 | -0.29 | 0.03 | -0.21 | -0.29 | 0.52 | 0.13 | -0.33 | -0.31 | 0.35 | 0.82 |
| *OsUBC11* | Os01g62244 | 1.01 | 0.70 | 0.45 | -1.15 | 0.71 | 1.52 | 0.74 | 0.26 | 0.04 | 0.26 | -1.12 | -2.17 | 0.44 | -0.6 | -0.03 | 0.74 | 1.47 |

YR, roots of 7-day-old seedlings; ML, mature leaves, YL, leaves of 7-day-old seedlings; SAM, up to 0.5 mm, shoot apical meristem; P1, 0-3 cm panicles; P2, 3-5 cm panicles; P3, 5-10 cm panicles; P4, 10-15 cm panicles; P5, 15-22 cm panicles; P6, 22-30 cm panicles; St, stigma; Ov, ovule; S1, 0-2 DAP seeds; S2, 3-4 DAP seeds; S3, 5-10 DAP seeds; S4, 11-20 DAP seeds;S5, 21-29 DAP seeds.
