# Supplementary material for: Characterization of the Ubiquitin-Conjugating Enzyme Gene Family in Rice and Evaluation of Expression Profiles under Abiotic Stresses and Hormone Treatments
Source: PLoS One. 2015 Apr 22;10(4):e0122621. doi: 10.1371/journal.pone.0122621 (PMC4406754; doi:10.1371/journal.pone.0122621)
Supplement: S3 Table — (DOC) [file pone.0122621.s009.doc]

**Table S3. The MPSS analysis of *OsUBC*** genes.

| **Gene** | **Locus** | **NYR** | **NST** | **NYL** | **NME** | **NPO** | **NIP** | **NCA** | **Max** |
| --- | --- | --- | --- | --- | --- | --- | --- | --- | --- |
| *OsUBC1* | LOC_Os10g39120 | 286 | 101 | 221 | 171 | 10 | 191 | 151 | 286 |
| *OsUBC2* | LOC_Os03g03130 | 342 | 248 | 425 | 42 | 210 | 269 | 349 | 425 |
| *OsUBC3* | LOC_Os04g49130 | 14 | 4 | 0 | 0 | 0 | 37 | **293** | 293 |
| *OsUBC4* | LOC_Os10g11260 | 209 | 422 | 335 | 296 | 119 | 313 | 211 | 422 |
| *OsUBC5* | LOC_Os08g28680 | 94 | 35 | 239 | 24 | 99 | 117 | 130 | 239 |
| *OsUBC6* | LOC_Os09g15320 | 6 | 5 | 0 | 15 | 12 | 14 | 22 | 22 |
| *OsUBC7* | LOC_Os07g07240 | 315 | 330 | 344 | 168 | 504 | 564 | 149 | 564 |
| *OsUBC8* | LOC_Os05g08960 | 0 | 141 | 218 | 172 | 113 | 127 | 74 | 218 |
| *OsUBC9* | LOC_Os03g57790 | 1458 | 1196 | 1385 | 727 | 1801 | 455 | 786 | 1801 |
| *OsUBC10* | LOC_Os10g31000 | 130 | 215 | 313 | 343 | 68 | 189 | 266 | 343 |
| *OsUBC11* | LOC_Os01g62244 | 0 | 37 | 97 | 104 | 44 | 1 | 45 | 104 |
| *OsUBC12* | LOC_Os05g38550 | 0 | 1 | 1 | 1 | 8 | 9 | 7 | 9 |
| *OsUBC13* | LOC_Os02g02830 | 18 | 0 | **126** | 0 | 0 | 0 | 0 | 126 |
| *OsUBC14* | LOC_Os01g46926 | 641 | 251 | 399 | 0 | 158 | 480 | 425 | 641 |
| *OsUBC15* | LOC_Os02g16040 | 0 | 148 | 20 | 1 | 66 | 98 | 0 | 148 |
| *OsUBC16* | LOC_Os04g57220 | 2006 | 1113 | 2351 | 1099 | 1779 | 1521 | 8393 | 8393 |
| *OsUBC17* | LOC_Os06g30970 | 4 | 6 | 74 | 0 | 0 | 6 | 98 | 98 |
| *OsUBC18* | LOC_Os09g12230 | 0 | 3 | 23 | 0 | 1 | 0 | 1 | 23 |
| *OsUBC22* | LOC_Os01g60360 | 0 | 0 | 0 | 54 | 0 | 0 | 0 | 54 |
| *OsUBC23* | LOC_Os01g60410 | 442 | 248 | 431 | 154 | **1060** | 457 | 398 | 1060 |
| *OsUBC25* | LOC_Os03g47770 | 19 | 72 | 93 | 36 | 214 | 56 | 105 | 214 |
| *OsUBC26* | LOC_Os12g44000 | 31 | 36 | 40 | 94 | 21 | 8 | 24 | 94 |
| *OsUBC27* | LOC_Os01g16650 | 0 | 5 | 0 | 0 | 2 | 0 | 0 | 5 |
| *OsUBC32* | LOC_Os02g42314 | 32 | 38 | 34 | 45 | 0 | 37 | 59 | 59 |
| *OsUBC33* | LOC_Os06g45000 | 24 | 0 | 26 | 0 | 38 | 12 | 20 | 38 |
| *OsUBC34* | LOC_Os01g03520 | 9 | 47 | 0 | 27 | 95 | 1 | 31 | 95 |
| *OsUBC35* | LOC_Os05g48390 | 527 | 12 | 133 | 7 | 74 | 50 | 0 | 527 |
| *OsUBC36* | LOC_Os05g06120 | - | - | - | - | - | - | - | - |
| *OsUBC37* | LOC_Os01g13280 | 0 | 0 | 0 | 0 | **17** | 0 | 0 | 17 |
| *OsUBC39* | LOC_Os01g48580 | - | - | - | - | - | - | - | - |
| *OsUBC40* | LOC_Os09g12310 | 16 | 0 | 0 | 0 | 0 | **99** | 0 | 99 |
| *OsUBC41* | LOC_Os05g48380 | 5 | 2 | 40 | 23 | 383 | 59 | 83 | 383 |
| *OsUBC42* | LOC_Os01g13170 | 3 | 3 | 46 | 12 | 99 | 0 | 18 | 99 |
| *OsUBC43* | LOC_Os05g14300 | 0 | 2 | 23 | 7 | **107** | 10 | 31 | 107 |
| *OsUBC44* | LOC_Os01g70140 | 124 | 186 | 28 | 121 | 140 | 169 | 151 | 186 |
| *OsUBC45* | LOC_Os03g19500 | 2 | 0 | 62 | 19 | 35 | 54 | 15 | 62 |
| *OsUBC46* | LOC_Os06g09330 | 656 | 224 | 175 | 105 | 0 | 142 | 94 | 656 |
| *OsUBC47* | LOC_Os01g48280 | 352 | 183 | 401 | 182 | 439 | 250 | 439 | 439 |
| *OsUBC48* | LOC_Os01g42040 | - | - | - | - | - | - | - | - |

Underlined and bold indicated specific expression; underlined indicated abundant expression; –, no expressed signatures. NYR, 14 d young roots; NST, 60 d stem; NYL, 14 d young leaves; NME, 60 d meristem tissue; NPO, mature pollen; NIP, 90 d immature panicle; NCA, 35 d callus; Max, maximum value.

Differential expression abundances are displayed by the number of tags (tpm, transcripts per million): tpm values <50 are considered low abundance, tpm values of 50 to 500 are considered moderate, and tpm values >500 tpm are considered as strong expression.
