# Supplementary material for: Characterization of the Ubiquitin-Conjugating Enzyme Gene Family in Rice and Evaluation of Expression Profiles under Abiotic Stresses and Hormone Treatments
Source: PLoS One. 2015 Apr 22;10(4):e0122621. doi: 10.1371/journal.pone.0122621 (PMC4406754; doi:10.1371/journal.pone.0122621)
Supplement: S4 Table — (DOC) [file pone.0122621.s010.doc]

**Table S4. Data for expession comparison of *OsUBCs* and *AtUBCs*** **in Fig.8**.

| **Group** | **Gene** | **R** | **L** | **I** | **P** | **S** | **DSS** | **DSR** | **SSS** | **SSR** | **CSS** | **CSR** |
| --- | --- | --- | --- | --- | --- | --- | --- | --- | --- | --- | --- | --- |
| 1 | ***OsUBC11*** | 1.94 | 1.57 | 2.77 | 44 | 2.67 | 0.75 | | 0.74 | | 1.11 | |
|  | ***OsUBC12*** | 1.72 | 2.18 | 2.40 | 8.00 | 2.58 | 0.77 | | 0.87 | | 1.02 | |
|  | ***AtUBC7*** | 0.42 | 0.29 | 0.68 | 0.74 | 0.38 | 0.78 | 0.84 | 1.30 | 0.70 | 0.87 | 1.06 |
|  | ***AtUBC13*** | 0.41 | 0.23 | 0.72 | 0.24 | 0.36 | 1.05 | 1.02 | 1.06 | 0.45 | 0.91 | 0.93 |
|  | ***AtUBC14*** | 0.48 | 0.37 | 0.58 | 1.96 | 0.39 | 0.64 | 0.89 | 0.73 | 0.68 | 0.87 | 1.04 |
| 1 | ***AtUBC1*** | 2.17 | 1.32 | 1.97 | 1.32 | 1.70 | 1.09 | 1.10 | 1.04 | 0.65 | 0.82 | 0.83 |
|  | ***OsUBC7*** | 2.07 | 1.52 | 1.79 | 504 | 1.89 | 0.93 | | 0.98 | | 1.01 | |
|  | ***OsUBC8*** | 1.67 | 1.12 | 1.20 | 113 | 1.25 | 0.57 | | 0.61 | | 1.01 | |
|  | ***AtUBC2*** | 2.43 | 1.45 | 2.97 | 2.61 | 3.93 | 1.32 | 0.90 | 0.98 | 0.68 | 0.47 | 0.64 |
|  | ***AtUBC3*** | 0.42 | 0.35 | 0.59 | 0.04 | 0.70 | 0.87 | 0.94 | 1.10 | 1.08 | 1.03 | 0.88 |
|  | ***OsUBC9*** | 2.70 | 4.84 | 3.79 | 1801 | 5.29 | 1.00 | | 0.99 | | 1.32 | |
| 1 | ***AtUBC12*** | 0.02 | 0.02 | 0.02 | 0.07 | 0.06 | 0.63 | 1.09 | 0.79 | 1.3 | 0.67 | 1.16 |
|  | ***OsUBC18*** | 1.73 | 0.44 | 0.35 | 1.00 | 0.33 | 0.43 | | 0.56 | | 0.97 | |
|  | ***AtUBC8*** | 4.22 | 1.55 | 3.17 | 2.68 | 2.49 | 0.80 | 0.79 | 1.13 | 0.57 | 0.86 | 0.86 |
|  | ***AtUBC9*** | 5.72 | 4.24 | 6.58 | 13.70 | 9.67 | 0.89 | 0.90 | 1.13 | 0.77 | 1.04 | 0.90 |
|  | ***AtUBC10*** | 5.72 | 4.24 | 6.58 | 13.70 | 9.67 | 0.89 | 0.90 | 1.13 | 0.77 | 1.04 | 0.90 |
|  | ***AtUBC28*** | 4.21 | 2.09 | 3.6 | 11.6 | 4.05 | 0.67 | 0.85 | 0.89 | 0.62 | 0.74 | 0.91 |
|  | ***OsUBC16*** | 3.33 | 2.46 | 6.93 | 1779 | 9.46 | 1.11 | | 1.08 | | 1.26 | |
|  | ***OsUBC23*** | 4.43 | 2.99 | 5.00 | 1060 | 6.73 | 0.77 | | 0.82 | | 0.94 | |
|  | ***AtUBC11*** | 0.29 | 0.13 | 1.36 | 0.25 | 1.98 | 1.17 | 0.9 | 1.19 | 0.59 | 0.79 | 0.94 |
|  | ***OsUBC15*** | 1.23 | 0.49 | 1.26 | 66 | 1.78 | 1.88 | | 1.49 | | 1.00 | |
|  | ***OsUBC17*** | 0.09 | 0.72 | 0.22 | 0 | 0.58 | 1.59 | | 1.24 | | 0.88 | |
|  | ***OsUBC22*** | × | × | × | 0 | × | × | | × | | × | |
|  | ***OsUBC14*** | × | × | × | 158 | × | × | | × | | × | |
|  | ***AtUBC30*** | 0.57 | 0.23 | 2.39 | 1.66 | 4.08 | 0.94 | 0.94 | 1.34 | 0.9 | 0.89 | 1.19 |
|  | ***AtUBC29*** | 0.77 | 0.11 | 1.42 | 0.13 | 0.39 | 1.03 | 0.94 | 1.31 | 1 | 1.02 | 1.51 |
|  | ***OsUBC13*** | 0.27 | 0.95 | 0.17 | 0 | 0.57 | 1.45 | | 1.34 | | 0.96 | |
| 1 | ***OsUBC47*** | 1.84 | 2.07 | 1.44 | 439 | 1.61 | 1.15 | | 0.97 | | 1.05 | |
|  | ***AtUBC35*** | 1.7 | 1.26 | 2.87 | 0.46 | 1.91 | 0.92 | 0.89 | 0.98 | 0.61 | 0.8 | 0.88 |
|  | ***AtUBC36*** | 1.7 | 1.26 | 2.87 | 0.46 | 1.91 | 0.92 | 0.89 | 0.98 | 0.61 | 0.8 | 0.88 |
| 1 | ***OsUBC48*** | 0.05 | 0.02 | 0.14 | × | 0.05 | 0.55 | | 0.6 | | 0.74 | |
|  | ***AtUBC37*** | 0.08 | 0.09 | 0.07 | 0.06 | 0.06 | 1.03 | 1.02 | 1.4 | 0.84 | 0.94 | 0.73 |
| 1 | ***OsUBC45*** | 0.79 | 1.23 | 1.15 | 35 | 1.62 | 1.52 | | 1.15 | | 0.97 | |
|  | ***AtUBC32*** | 0.26 | 0.23 | 1.51 | 0.36 | 1.32 | 0.86 | 1.25 | 3.02 | 1.13 | 0.9 | 1.22 |
|  | ***OsUBC46*** | 0.37 | 0.64 | 0.85 | 0 | 0.90 | 1.24 | | 1.14 | | 1.11 | |
|  | ***AtUBC33*** | 0.40 | 0.37 | 0.93 | 0.86 | 0.89 | 1.04 | 0.99 | 1.16 | 1 | 0.99 | 0.88 |
|  | ***AtUBC34*** | 0.40 | 0.37 | 0.93 | 0.86 | 0.89 | 1.04 | 0.99 | 1.16 | 1 | 0.99 | 0.88 |

R, root; L, leaf; I, inflorescence; P, pollen; S, silique or seed; DSS and DSR; drought stressed shoot and root; SSS and SSR, salt stressed shoot and root; CSS and CSR, cold stressed shoot and root. ×, no expressed signatures.
