# Supplementary material for: Characterization of the Ubiquitin-Conjugating Enzyme Gene Family in Rice and Evaluation of Expression Profiles under Abiotic Stresses and Hormone Treatments
Source: PLoS One. 2015 Apr 22;10(4):e0122621. doi: 10.1371/journal.pone.0122621 (PMC4406754; doi:10.1371/journal.pone.0122621)
Supplement: S5 Table — (DOC) [file pone.0122621.s011.doc]

## Table S5. Primers used in qRT-PCR of rice *UBC* genes.

| **Genes** | **Primers used in qRT-PCR(5′→3′)** | |
| --- | --- | --- |
| *OsUBC1* | Forwards： | CTACCCTCTTACCCTTCACTTC |
|  | Reverse： | GATAGCAGGTCTCCAGCCAC |
| *OsUBC2* | Forwards： | CAGTATCCTCCGATTGTCT |
|  | Reverse： | AAACTCCGAAAGCACCCT |
| *OsUBC3* | Forwards： | GATGATCCAAACCCTAACTC |
|  | Reverse： | CAACTCGGCAACTCAATACA |
| *OsUBC4* | Forwards： | ACTCTTCTAGCGATTGTG |
|  | Reverse： | CTCTTGGGTAGGTTTAGC |
| *OsUBC5* | Forwards： | GGCTGGAGGCTATGTTGGT |
|  | Reverse： | TCTTCGGATTGCGGGTTAT |
| *OsUBC6* | Forwards： | AAAGGGACAGAAGAAGGAA |
|  | Reverse： | TGAGGGTAAGTAGGAGGAAC |
| *OsUBC7* | Forwards： | ATTTCCCTCAACTATCAGC |
|  | Reverse： | GAGACTTAGAAGACGACCAG |
| *OsUBC8* | Forwards： | TGTTTCTCGGATGTTTCA |
|  | Reverse： | GCTTCAGAGTTGGCAGGT |
| *OsUBC9* | Forwards： | TCGGTTTGTCTCAAGGATG |
|  | Reverse： | CAGAGCAGGGACTGGATAG |
| *OsUBC10* | Forwards： | CTGCACTGTTGCGTTTC |
|  | Reverse： | ATCTGGGTTGCCGAGTA |
| *OsUBC11* | Forwards： | CTCCCAGGAAGGCTGAATA |
|  | Reverse： | AAAGAACGGACTGAAACCA |
| *OsUBC12* | Forwards： | AAGATAAGGAGCACAAGGG |
|  | Reverse： | CTCGGAGACAAGACCAAC |
| *OsUBC13* | Forwards： | AATCAGTCAATCACCAGCAT |
|  | Reverse： | ATTATTCCTCCTCACCAAGA |
| *OsUBC14* | Forwards： | GGATGGCGTCAAAGAGGATA |
|  | Reverse： | AGGAGGGAAGTGGATGGTAA |
| *OsUBC15* | Forwards： | TATCCATTCAAACCGCCTAA |
|  | Reverse： | ATCCGTCAACAGCGAGCAAA |
| *OsUBC16* | Forwards： | TGCCGGAGATTGCTCACA |
|  | Reverse： | AACGGACTTCACAAGGTAGGG |
| *OsUBC17* | Forwards： | ACCTGCGTGCTAATCTGG |
|  | Reverse： | GGAACTAGCGGGTCGTCT |
| *OsUBC18* | Forwards： | TATCACCCAAACATCAACTCC |
|  | Reverse： | GCTGCTAACCCATTGCGTAT |
| *OsUBC22* | Forwards： | TGGCAGGCAACCATTAT |
|  | Reverse： | AGTGAGCAGCGAGCAGA |
| *OsUBC23* | Forwards： | GGATGATGAAACCCACAA |
|  | Reverse： | CAGCAACAAATCGGAGGA |
| *OsUBC25* | Forwards： | AATGATCGCTATGTCAGGAA |
|  | Reverse： | CATGTAAAGGTAGGGTGGC |
| *OsUBC26* | Forwards： | AACGCCCTCAAGACAATG |
|  | Reverse： | ACAAACCAACCCAGCAGT |
| *OsUBC27* | Forwards： | ACAAGCCCACCGCGTAGAAG |
|  | Reverse： | CAATCCGACTCGCACAACAAT |
| *OsUBC32* | Forwards： | TCAGGCGACATCAGAGGC |
|  | Reverse： | AGGGCATACATCCATAAGAGTG |
| *OsUBC33* | Forwards： | TCCCTCAGTCTCCTCCTA |
|  | Reverse： | GAAGGCATCTCACTACCAG |
| *OsUBC34* | Forwards： | TAAGCCTTCATTACACCCA |
|  | Reverse： | AAGCGAACAACAACATCTC |
| *OsUBC35* | Forwards： | CATGGGCAGGAACTGGTAG |
|  | Reverse： | CAGCCTCATTGAAGTAAGGTTT |
| *OsUBC36* | Forwards： | CCGCCTGCGAGTCGTACCT |
|  | Reverse： | CGCCGAACCGCTCACACC |
| *OsUBC37* | Forwards： | GACGCATACATATCTGGC |
|  | Reverse： | CTGCTGGTACACTCAACG |
| *OsUBC39* | Forwards： | TGTCACTTGCCGATTACC |
|  | Reverse： | CAGCAGAAGTTTGTCCCT |
| *OsUBC40* | Forwards： | AAAAGCTGCTCAGCGAACT |
|  | Reverse： | TAGCCTCAATGTGGTGTCC |
| *OsUBC41* | Forwards： | GCTCCAGCAGACACCACAT |
|  | Reverse： | AGGCTACTTCCAGGAAACG |
| *OsUBC42* | Forwards： | GGCAGGTAGTGGTTGTGA |
|  | Reverse： | CGCCGAAGTGAGTAGAGC |
| *OsUBC43* | Forwards： | GTTGGGAAGACAGGGAAAG |
|  | Reverse： | CACTGGAGGAGTAGATGGATA |
| *OsUBC44* | Forwards： | AGTGTCAATGGCGACGAGAA |
|  | Reverse： | ACTTAAATGGCAGAACAGGAG |
| *OsUBC45* | Forwards： | CAATCCAGAAGCCAAAGCA |
|  | Reverse： | CATAGCAGCCGCTCAGACA |
| *OsUBC46* | Forwards： | ATTTCCACCTGATTACCCTT |
|  | Reverse： | ATGCTTGCCACAGACCAC |
| *OsUBC47* | Forwards： | GGAAAGCCAATGAAGCAGAA |
|  | Reverse： | GGGTAAAGGGAACCAACCAC |
| *OsUBC48* | Forwards： | CATAGGCTACCGTGTAAAGTC |
|  | Reverse： | AGGGCAACATAAAGATAAGAAC |
| UBQ5 | Forwards： | ACCACTTCGACCGCCACTACT |
|  | Reverse： | ACGCCTAAGCCTGCTGGTT |
