# Supplementary material for: Characterization of the Ubiquitin-Conjugating Enzyme Gene Family in Rice and Evaluation of Expression Profiles under Abiotic Stresses and Hormone Treatments
Source: PLoS One. 2015 Apr 22;10(4):e0122621. doi: 10.1371/journal.pone.0122621 (PMC4406754; doi:10.1371/journal.pone.0122621)
Supplement: S6 Table — (DOC) [file pone.0122621.s012.doc]

**Table S6. Data for expession comparison of *OsUBCs* and *AtUBCs* in Fig.S6.**

| **Group** | **Gene** | **R** | **L** | **I** | **P** | **S** | **DSS** | **DSR** | **SSS** | **SSR** | **CSS** | **CSR** |
| --- | --- | --- | --- | --- | --- | --- | --- | --- | --- | --- | --- | --- |
| I | ***OsUBC1*** | 1.88 | 1.70 | 1.46 | 10 | 1.88 | 0.75 | | 0.88 | | 0.87 | |
|  | ***OsUBC2*** | 1.81 | 1.74 | 1.99 | 210 | 2.03 | 0.59 | | 0.86 | | 0.98 | |
|  | ***OsUBC3*** | 0.10 | 0.01 | 0.34 | 0 | 0.09 | 1.00 | | 1.08 | | 1.36 | |
| 1 | ***OsUBC27*** | 0.18 | 0.01 | 0.10 | 2.00 | 0.07 | 0.48 | | 0.70 | | 0.82 | |
|  | ***AtUBC19*** | 0.37 | 0.47 | 0.80 | 0.33 | 0.97 | 0.88 | 0.84 | 0.84 | 0.62 | 0.98 | 0.73 |
|  | ***AtUBC20*** | 0.61 | 0.47 | 0.67 | 0.85 | 0.37 | 1.03 | 0.78 | 1.01 | 0.47 | 0.77 | 0.93 |
| 1 | ***OsUBC32*** | × | × | × | 0 | × | × | | × | | × | |
|  | ***AtUBC21*** | 0.67 | 0.48 | 0.75 | 0.31 | 0.75 | 0.79 | 0.92 | 1.02 | 0.68 | 0.83 | 0.87 |
| 1 | ***AtUBC15*** | 0.20 | 0.15 | 0.88 | 0.96 | 0.22 | 1.04 | 1.02 | 1.16 | 0.71 | 1.02 | 0.89 |
|  | ***AtUBC16*** | 0.23 | 0.02 | 0.31 | 0 | 0.45 | 1.01 | 1.02 | 1.05 | 1.29 | 1.31 | 1.28 |
|  | ***AtUBC17*** | 0.21 | 0.16 | 0.68 | 0.03 | 0.18 | 0.68 | 1.24 | 0.32 | 0.39 | 0.98 | 1.17 |
|  | ***AtUBC18*** | 0.38 | 0.19 | 0.42 | 0.10 | 0.74 | 0.86 | 0.86 | 1.53 | 0.8 | 1.12 | 0.80 |
|  | ***OsUBC25*** | 0.53 | 0.60 | 0.65 | 214 | 0.79 | 0.71 | | 0.71 | | 0.77 | |
|  | ***OsUBC26*** | 0.69 | 0.57 | 0.57 | 21 | 0.50 | 0.72 | | 0.79 | | 0.93 | |
| II | ***OsUBC4*** | 2.29 | 1.87 | 2.3 | 119 | 2.58 | 0.82 | | 0.94 | | 1.03 | |
|  | ***OsUBC5*** | 0.94 | 1.4 | 1.66 | 99 | 1.70 | 0.56 | | 0.82 | | 0.87 | |
|  | ***OsUBC6*** | 0.08 | 0.06 | 0.36 | 12 | 3.81 | 1.78 | | 2.15 | | 1.13 | |
| 1 | ***OsUBC10*** | 2.67 | 1.36 | 1.46 | 68 | 4.92 | 1.04 | | 1.07 | | 0.97 | |
|  | ***AtUBC4*** | 0.41 | 0.31 | 0.69 | 0.31 | 1.22 | 0.90 | 1.12 | 1.20 | 0.62 | 0.70 | 0.98 |
|  | ***AtUBC5*** | 0.60 | 0.31 | 1.68 | 0.02 | 0.90 | 0.71 | 0.83 | 1.07 | 0.54 | 0.48 | 0.97 |
|  | ***AtUBC6*** | 0.20 | 0.18 | 1.44 | 0.21 | 0.40 | 1.39 | 1.05 | 0.83 | 1.1 | 0.9 | 1.27 |
| 1 | ***OsUBC33*** | 0.43 | 0.14 | 0.45 | 38 | 0.45 | 0.71 | | 0.64 | | 0.93 | |
|  | ***AtUBC22*** | 1.33 | 0.70 | 1.2 | 0.76 | 0.94 | 0.98 | 0.8 | 1.17 | 0.58 | 0.73 | 0.96 |
| 1I | ***AtUBC31*** | 0.01 | 0 | 0.05 | 0.01 | 0.30 | 0.42 | 1.49 | 0.26 | 0.64 | 0.32 | 0.97 |
| 1 | ***OsUBC44*** | 0.73 | 0.41 | 0.73 | 140 | 0.86 | 0.58 | | 0.82 | | 1.14 | |
|  | ***AtUBC27*** | 0.52 | 0.56 | 0.78 | 0.35 | 0.77 | 0.82 | 0.92 | 0.95 | 0.75 | 0.87 | 0.93 |
| 1I | ***OsUBC34*** | 0.32 | 0.32 | 0.33 | 95 | 0.52 | 0.88 | | 1.03 | | 0.83 | |
|  | ***AtUBC23*** | 0.16 | 0.11 | 0.19 | 0.26 | 0.22 | 0.91 | 1 | 0.87 | 0.71 | 1.12 | 0.97 |
|  | ***AtUBC24*** | × | × | × | × | × | × | × | × | × | × | × |
|  | ***OsUBC35*** | 1.02 | 0.64 | 0.35 | 74 | 0.40 | 0.34 | | 0.63 | | 0.90 | |
|  | ***OsUBC36*** | 0.02 | 0.02 | 0.04 | × | 0.05 | 1.63 | | 2.01 | | 1.62 | |
|  | ***AtUBC25*** | 0.05 | 0.04 | 0.08 | 0.09 | 0.10 | 1.25 | 0.94 | 1.15 | 0.81 | 0.86 | 0.91 |
|  | ***AtUBC26*** | 0.02 | 0.01 | 0.02 | 0.05 | 0.03 | 0.42 | 1.90 | 0.82 | 1.23 | 0.75 | 1.12 |
|  | ***OsUBC42*** | 0.20 | 0.68 | 0.72 | 99 | 0.62 | 0.77 | | 1.32 | | 1.11 | |
|  | ***OsUBC43*** | 0.04 | 0.07 | 0.04 | 107 | 0.09 | 1.08 | | 1.31 | | 2.53 | |
|  | ***OsUBC37*** | 0.04 | 0.04 | 0.12 | 17 | 0.16 | 2.21 | | 2.13 | | 1.11 | |
|  | ***OsUBC39*** | 0.01 | 0.01 | 0.01 | × | 0.07 | 1.53 | | 1.58 | | 0.92 | |
|  | ***OsUBC40*** | 0.01 | 0.02 | 0.02 | 0 | 0.02 | 1.05 | | 1.45 | | 1.28 | |
|  | ***OsUBC41*** | 0.20 | 0.44 | 0.46 | 383 | 0.81 | 0.95 | | 1.12 | | 1.24 | |

R, root; L, leaf; I, inflorescence; P, pollen; S, silique or seed; DSS and DSR; drought stressed shoot and root; SSS and SSR, salt stressed shoot and root; CSS and CSR, cold stressed shoot and root. ×, no expressed signatures.
